# Incidence of chemotherapy‐related cardiac dysfunction in cancer patients

**DOI:** 10.1002/clc.24269

**Published:** 2024-04-18

**Authors:** Hai‐Wei Deng, Rui Fan, Yuan‐Sheng Zhai, Jie Li, Zhi‐Bin Huang, Long‐Yun Peng

**Affiliations:** ^1^ Department of Cardiology, The First Affiliated Hospital Sun Yat‐Sen University Guangzhou China; ^2^ Key Laboratory on Assisted Circulation Ministry of Health Guangzhou China; ^3^ Department of Medical Ultrasonics, The First Affiliated Hospital Sun Yat‐Sen University Guangzhou China

**Keywords:** cancer, cardiac dysfunction, chemotherapy

## Abstract

**Background:**

Cancer patients are increasingly affected by chemotherapy‐related cardiac dysfunction. The reported incidence of this condition vary significantly across different studies.

**Hypothesis:**

A better comprehensive understanding of chemotherapy‐related cardiac dysfunction incidence in cancer patients is imperative. Therefore, we performed a meta‐analysis to establish the overall incidence of chemotherapy‐related cardiac dysfunction in cancer patients.

**Methods:**

We searched articles in PubMed and EMBASE from database inception to May 1, 2023. Studies that reported the incidence of chemotherapy‐related cardiac dysfunction in cancer patients were included.

**Results:**

A total of 53 studies involving 35 651 individuals were finally included in the meta‐analysis. The overall pooled incidence of chemotherapy‐related cardiac dysfunction in cancer patients was 63.21 per 1000 person‐years (95% CI: 57.28−69.14). The chemotherapy‐related cardiac dysfunction incidence increased steeply within half a year of cancer chemotherapy. Also, the trend of chemotherapy‐related cardiac dysfunction incidence appeared to have plateaued after a longer duration of follow‐up. In addition, chemotherapy‐related cardiac dysfunction incidence rates are significantly higher among patients with age ≥50 years versus patients with age <50 years (99.96 vs. 34.48 per 1000 person‐years). The incidence rate of cardiac dysfunction was higher among breast cancer patients (72.97 per 1000 person‐years), leukemia patients (65.21 per 1000 person‐years), and lymphoma patients (55.43 per 1000 person‐years).

**Conclusion:**

Our meta‐analysis unveiled a definitive overall incidence rate of chemotherapy‐related cardiac dysfunction in cancer patients. In addition, it was found that the risk of developing this condition escalates within the initial 6 months postchemotherapy, subsequently tapering off to become statistically insignificant after a duration of 6 years.

## Graphical abstract

we performed a meta‐analysis to establish the overall incidence of chemotherapy‐related cardiac dysfunction in cancer patients. The overall pooled incidence of chemotherapy‐related cardiac dysfunction in cancer patients was 63.21 per 1000 person‐years (95% CI: 57.28−69.14).

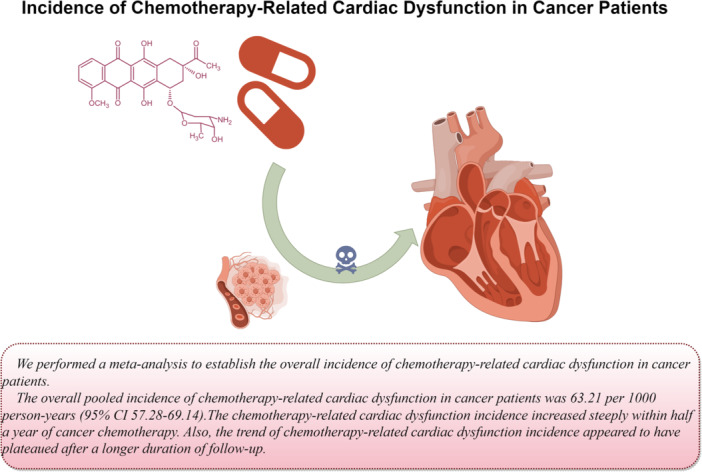

**Abbreviations**
CI: confidence Interval
LVEF: left ventricular ejection fraction
RCT: random controlled trial
ROS: reactive oxygen species

## 1 INTRODUCTION

Cancer significantly contributes to the global burden of disease and is among the leading etiologies of death.^1^ The deployment of anticancer drugs has become increasingly prevalent in the therapeutic arsenal against cancer, and advanced therapeutic agents are consistently emerging from research and development efforts. Thus, the mortality rate of cancer has continued to decline despite its high incidence, and as a result, the number of cancer survivors has increased.^2^ However, there are many side effects associated with conventional chemotherapy and novel targeted cancer treatments, including cardiotoxicity.^3^ What's more, it is likely that cardiotoxicity will exceed cancer itself in terms of its harmful effects. Patients are increasingly affected by chemotherapy‐related cardiotoxicity. It was found that cancer patients experience cardiotoxicity outcomes that contribute to 7%−27% of their cardiovascular mortality.^4^, ^5^ This may be due to a number of different factors related to therapy, but also likely due to patients with preexisting cardiovascular risk phenotypes.

In addition, cardiac dysfunction is widely recognized as one of the most prevalent and severe forms of cardiotoxicity, posing a significant risk to patients' cardiovascular health during cancer therapy.^6^ There were quite a few reports on the incidence of chemotherapy‐related cardiac dysfunction in cancer patients. However, owing to disparities in economic development and healthcare infrastructure globally, the reported overall incidence of chemotherapy‐related cardiac dysfunction is widely variable, ranging from 1.90 to 794.19 per 1000 person‐years.^7^, ^8^ Despite the substantial body of research that has already been conducted in this domain, many studies have involved a small sample size and there are still no large‐scale systematic studies of the incidence of chemotherapy‐related cardiac dysfunction in cancer patients have been published.

In light of this, a better comprehensive understanding of chemotherapy‐related cardiac dysfunction incidence in cancer patients is imperative. Therefore, we performed a meta‐analysis to establish the overall incidence of chemotherapy‐related cardiac dysfunction in cancer patients. The duration of follow‐up and the incidence of chemotherapy‐related cardiac dysfunction were examined. Also, we performed a further analysis based on the type of study, sample size, age, female proportion, location, the type of cancer, publication year, and the criterion of chemotherapy‐related cardiac dysfunction. These data have the potential to inform healthcare systems in crafting targeted interventions and evidence‐based guidelines to optimize patient care and mitigate the risks associated with chemotherapy‐related cardiac dysfunction.

## 2 METHODS

### 2.1 Search strategy and selection criteria

To conduct this meta‐analysis, two authors (H.‐W. D. and R. F.) independently searched PubMed and EMBASE from database inception to May 1, 2023, using the following text and keywords combination both as MeSH terms and text words: “cancer,” “neoplasms, “cardiotoxicity,” “tumor,” “carcinoma,” “heart failure,” “cardiac dysfunction,” “myocardial dysfunction,” and “left ventricular dysfunction.” The search was limited to humans, and no language restrictions were applied. Besides, to identify other relevant studies, we reviewed references from these studies.

For the purpose of this analysis, we established inclusion and exclusion criteria. The inclusion criteria were described as follows: (1) Original research articles containing data about the incidence of chemotherapy‐related cardiac dysfunction in cancer patients were either available or allowed for such calculations. (2) Studies included patients with a decreased left ventricular ejection fraction (LVEF). The following exclusion criteria were used to enhance the representativeness and reliability of our study: (1) The sample size in the study was less than 100. (2) Studies were categorized as case series, letters, reviews, commentary, or editorials. In instances where multiple studies addressed the same population or subpopulation, preference was given to the study that presented the most current or comprehensive data, and ensuring that our analysis was grounded in the latest and most informative findings available. To conduct our meta‐analysis, we followed the PRISMA (Preferred Reporting Items for Systematic Review and Meta‐Analysis) guidelines.

### 2.2 Data extraction and analysis

Two investigators (H.‐W. D. and R. F.) used a preconceived and standardized form to extract the relevant data. Initially, titles and abstracts of articles were screened, followed by full‐text articles. Quality review agreement between the two authors ranged from 88% to 100%. Discussions between the two authors or consultation with a third senior researcher resolved discrepancies. Based on the included studies, the following data were abstracted: first author, year of publication, region/country, data sources, study design, follow‐up data, age, the percentage of females, sample size, cancer type, treatment, cardiac dysfunction criterion, and outcomes. The main outcome of this analysis was the incidence of chemotherapy‐related cardiac dysfunction in cancer patients. The definitions of chemotherapy‐related cardiac dysfunction are different. To guarantee the quality of articles and minimize study population heterogeneity, cardiac dysfunction was defined as heart failure, and myocardial dysfunction with LVEF decreased in this study.

To summarize data from multiple studies, we used Stata software, version 15.0 (Stata Corp.). The incidence of chemotherapy‐related cardiac dysfunction in cancer patients was expressed as per 1000 person‐years of follow‐up with 95% confidence intervals (CIs). The variance‐stabilizing Freeman‐Tukey double‐arcsine transformation was used to calculate weighted meta‐analytic incidence estimates. As the study settings varied among populations, we used an inverse‐variance random‐effects model. *I* ^2^ statistics were used to measure heterogeneity, with *I* ^2^ of at least 50% indicating significant heterogeneity. Also, an analysis of the relationship between follow‐up years and incidence was conducted using a fractional polynomial model. In addition, the following covariates were examined in subgroup analyses to investigate sources of heterogeneity: the type of study, sample size, age, female proportion, study location, the type of cancer, publication year, and the criterion of cardiac dysfunction. *p* Values between subgroups were calculated using meta‐regression. We used Egger's test to assess for publication bias. All *p* values were from two‐sided tests, and results were deemed statistically significant at *p* < .05.

A protocol for the study was registered in the Prospective Register of Systematic Reviews (PROSPERO CRD42023394448) before data were extracted.

## 3 RESULTS

**Figure 1:**
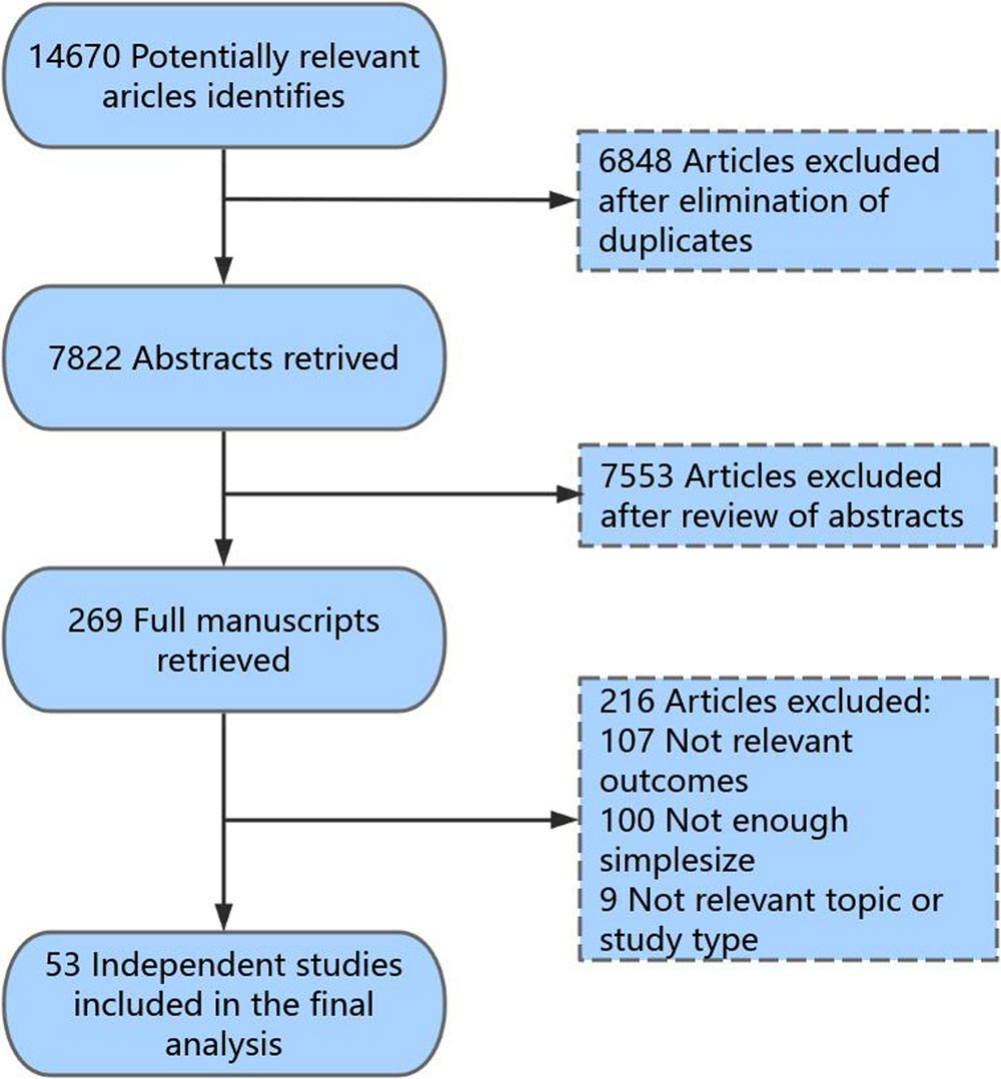
Flowchart of the selection of studies included in the meta‐analysis.

Based on our search method, we successfully identified 14 670 articles. Subsequent to the elimination of duplicate entries, a total of 7822 unique records remained for further screening. As a result of screening the titles and abstracts, we excluded 7553 ineligible records. In consequence, we retained and evaluated the full text of 269 publications. A total of 53 studies were finally included in the meta‐analysis (Figure 1).

In total, 35 651 individuals were examined (ranging from 100 to 4190 in each study and approximately 1994 from Oceania, 3657 from Asia, 5102 from America, and 15 510 from Europe). The temporal scope of the studies extended from the year 2002 to 2022, encompassing a two‐decade period. And various study designs were employed, comprising 11 randomized controlled trials (RCTs), 19 prospective cohort studies, and 23 retrospective reviews. Across all studies, duration of follow‐upranged from 0.24 to 9.23 years. When considering specific cancer types, breast cancer emerged as the most frequently researched. An overview of the study characteristics is provided in Table S1.

**Figure 2:**
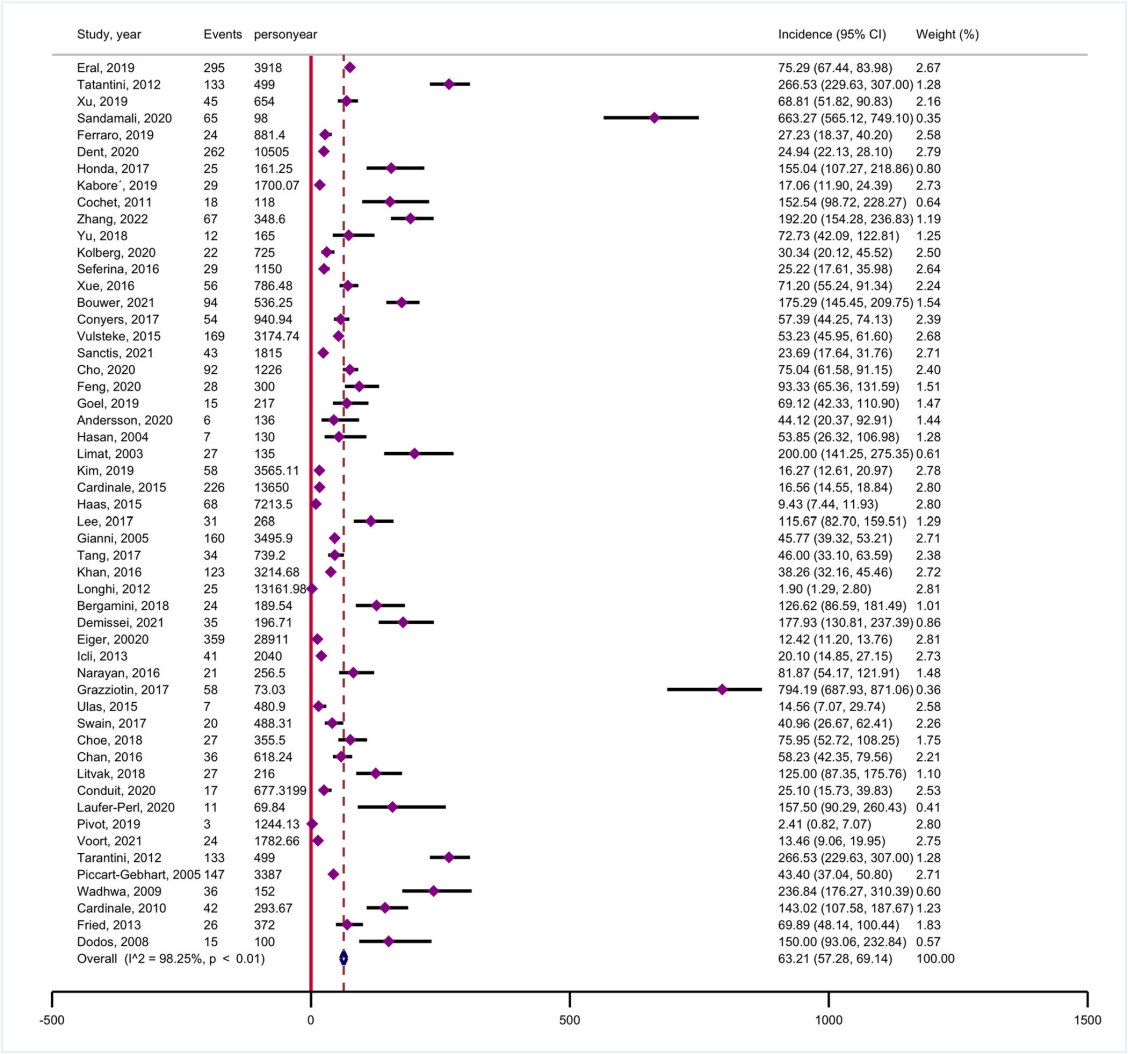
Forest plot showing the incidence of chemotherapy‐related cardiac dysfunction.

The overall pooled incidence of chemotherapy‐related cardiac dysfunction in cancer patients was 63.21 per 1000 person‐years (95% CI: 57.28−69.14) with very high between‐sample heterogeneity (*p* < .001; *I* ^2^ = 98.25%) (Figure 2). The incidence rates exhibited significant variation, ranging from 1.90 (95% CI: 1.29−2.80) to 794.19 (95% CI: 687.93−871.06) per 1000 person‐years, representing a nearly 418‐fold difference. The funnel plot is shown in Supporting Information S1: Figure 1. Egger's test indicated publication bias (*p* < .001).

**Figure 3:**
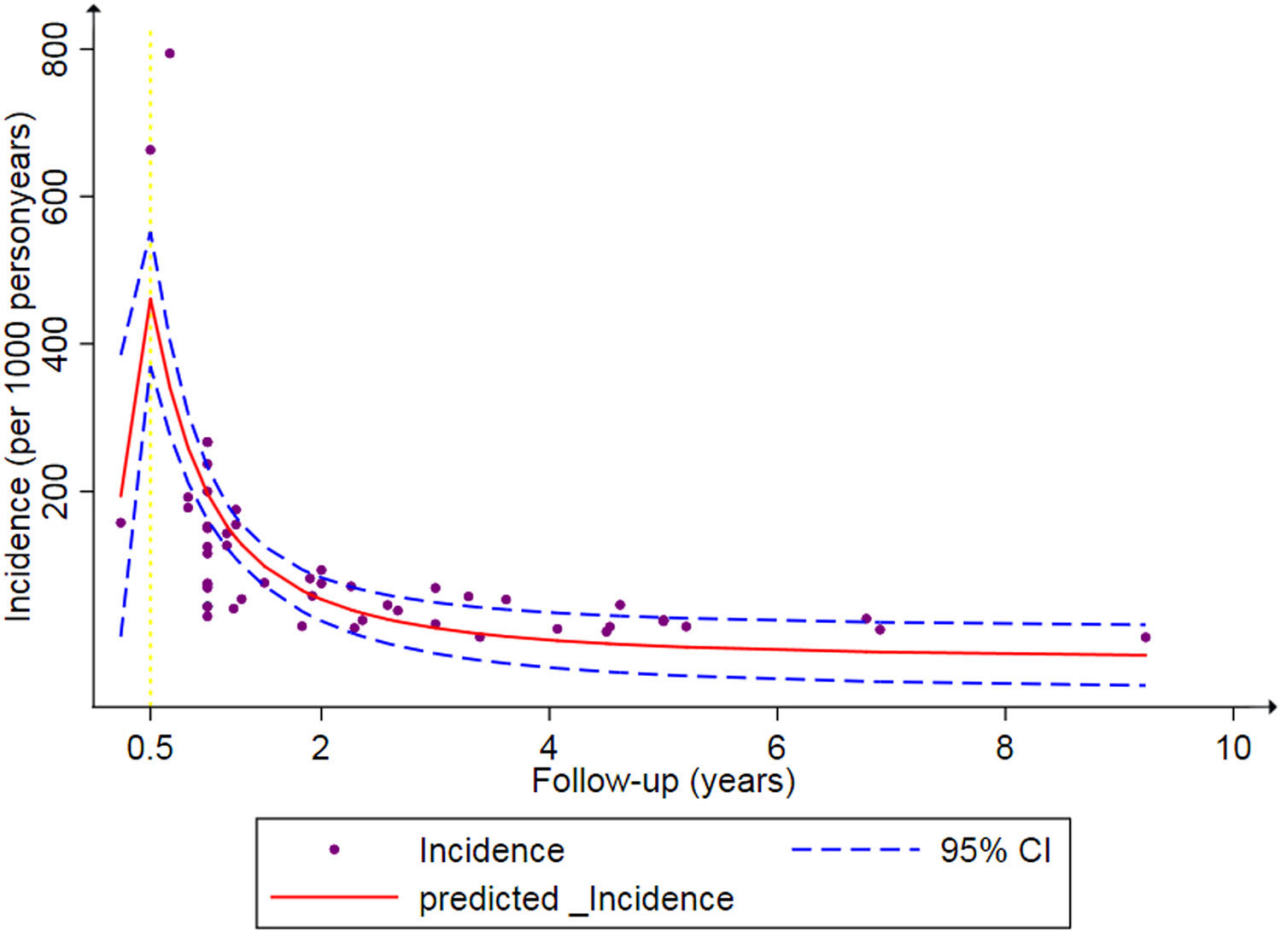
The relationship between chemotherapy‐related cardiac dysfunction incidence and follow‐up.

An assessment of the relationship between chemotherapy‐related cardiac dysfunction incidence and the duration of follow‐up was conducted as well. Five studies reported a follow‐up duration of less than 1 year, 41 studies covered a period of 1–5 years, and seven studies had a follow‐up period exceeding 5 years. According to the graph, chemotherapy‐related cardiac dysfunction incidence increased steeply within half a year of cancer chemotherapy. Also, the trend of chemotherapy‐related cardiac dysfunction incidence appeared to have plateaued after a longer duration of follow‐up (approximately >6 years) (Figure 3).

To explore the source of study heterogeneity, we stratified analyses by several of key study characteristics and clinical factors, including study design, sample size, age, female proportion, location, cancer type, publication year, and diagnostic criteria for cardiac dysfunction (Table 1 and Supporting Information S1: Figure 2–9). It is noteworthy that the observed findings suggest that the results are unlikely to be significantly influenced by factors such as study design, proportion of female participants, geographical location of the studies, publication year, and the criteria used to define cardiac dysfunction.

**Table 1:** Stratified analysis and heterogeneity analysis.

Factors stratified	Incidence (per 1000 person years [95% CI])	*p* Value
Type of study		
RCT	38.95 (29.73−48.17)	.104
Retrospective	85.15 (71.22−99.08)	
Prospective	93.56 (78.26−108.87)	
Sample size		
<1000	94.78 (83.09−106.46)	.002
≥1000	28.93 (20.77−37.09)	
Age (years)		
≥50	99.96 (87.13−112.80)	.048
<50	34.48 (24.96−44.00)	
Female proportion (%)		
≥50	69.39 (62.07−76.72)	.126
<50	48.27 (34.81−61.73)	
Location		
Europe	66.00 (54.94−77.06)	.499
Asia	135.85 (95.33−176.37)	
American	98.77 (78.69−118.85)	
Oceania	43.23 (28.57−57.89)	
Type of cancer		
Breast cancer	72.97 (65.01−80.93)	.022
Lymphoma	55.43 (33.55−77.32)	
Leukemia	65.21 (51.18−79.24)	
Other^a^	12.02 (4.06−19.97)	
Publication year		
<2008	55.60 (37.36−73.84)	.405
≥2008 and <2013	173.43 (56.90−289.96)	
≥2013 and <2018	55.91 (44.70−67.13)	
≥2018	62.71 (53.23−72.19)	
Criterion		
1^b^	38.72 (29.28−48.17)	.075
2^c^	63.55 (51.58−75.53)	
3^d^	102.03 (85.40−118.66)	

Abbreviations: LVEF, left ventricular ejection fraction; RCT, randomized controlled trial.

^a^ Including renal cell carcinoma, osteosarcoma, Ewing sarcoma, neuroblastoma, nephroblastoma, and so on.

^b^ Asymptomatic LVEF decreases ≥10% from baseline to below 50%; heart failure.

^c^ LVEF decreased by >10% from baseline to <50%.

d LVEF decrease not meet criteria 1 and 2.

Differences in cardiac dysfunction according to sample sizes were statistically significant. The incidence rate of studies with small sample sizes (<1000) was 94.78 (95% CI: 83.09−106.46) per 1000 person‐years, significantly higher than 28.93 (95% CI: 20.77−37.09) per 1000 person‐years of studies with large sample sizes (≥1000) (Supporting Information S1: Figure 3).

The baseline age was demonstrated in 47 of the identified studies. It ranged from 8.80 to 68.00 (mean: 51.45) years old. Chemotherapy‐related cardiac dysfunction incidence rates are significantly higher among patients with age ≥50 years versus patients with age <50 years (99.96 [95% CI: 87.13−112.80] vs. 34.48 [95% CI: 24.96−44.00] per 1000 person‐years) (Supporting Information S1: Figure 4).

In addition, the specific type of cancer under investigation may exert an influence on the study outcomes. The incidence rate of cardiac dysfunction was higher among breast cancer patients (72.97 [95% CI: 65.01−80.93] per 1000 person‐years), leukemia patients (65.21 [95% CI: 51.18−79.24] per 1000 person‐years), and lymphoma patients (55.43 [95% CI: 33.55−77.32] per 1000 person‐years) (Supporting Information S1: Figure 7).

Moreover, within the pool of included studies, breast cancer emerged as the most frequently examined cancer type (41 studies). It is well‐established that anthracyclines and trastuzumab are highly effective chemotherapeutic agents commonly used to treat breast cancer. The incidence of chemotherapy‐related cardiac dysfunction stratified according to breast cancer treatment was reported (Supporting Information S1: Figure 10). It was found that the incidence of cardiac dysfunction associated with anthracyclines was 49.93 (95% CI: 37.68−62.17) per 1000 person‐years, associated with trastuzumab was 117.22 (95% CI: 57.49−176.85) per 1000 person‐years, and associated with anthracyclines plus trastuzumab was 145.14 (95% CI: 109.33−180.96) per 1000 person‐years. However, the difference was not statistically significant (*p* = .310).

## 4 DISCUSSION

It is the most comprehensive and up‐to‐date systematic review we have ever found regarding the incidence of chemotherapy‐related cardiac dysfunction in cancer patients, summarizing estimates from 53 original studies. Based on the present meta‐analysis, there is an incidence rate of 63.21 per 1000 person‐years of chemotherapy‐related cardiac dysfunction in cancer patients. The risk of developing chemotherapy‐related cardiac dysfunction was more common in patients over 50 years of age and in patients with breast cancer. Furthermore, the risk of chemotherapy‐related cardiac dysfunction in cancer patients fluctuates throughout the course of the disease. The risk tended to increase during the initial stage (half a year) after chemotherapy, and then declined and became nonsignificant at 6 years onwards.

Numerous investigations have been conducted on chemotherapy‐related cardiac dysfunction in cancer patients. The incidence of chemotherapy‐related cardiac dysfunction varied extremely between studies. Eiger et al. reported that with 6.9 years of median follow‐up and 4190 breast cancer patients, cardiac dysfunction was observed in 359 (12.42 per 1000 person‐years).^9^ In a prospective analysis with a small sample size (100) in Germany, 10 participants experienced cardiac dysfunction (63.21 per 1000 person‐years).^10^ Analysis of a Chinese RCT showed that the incidence of chemotherapy‐related cardiac dysfunction in lymphoma patients was 71.20 per 1000 person‐years.^11^ A multitude of factors can influence the outcomes. It has been observed that the majority of prior research has concentrated solely on populations with a homogenous demographic profile. Thus, to provide a comprehensive analysis, we included data from different cancers, sexes, ages, regions, and ethnicities.

Chemotherapy‐related cardiac dysfunction risks vary widely among patients based on the cancer's type. The risk of developing cardiac dysfunction was higher in patients with breast cancer, leukemia, and lymphoma. Possibly, this could be attributed to their frequent treatment with anthracyclines. It is well known that anthracyclines have been highly effective against a wide variety of cancers, including leukemia, breast cancer, and lymphomas.^12^ However, administration of anthracyclines, such as doxorubicin or epirubicin, is associated with an increased risk of heart failure and left ventricular dysfunction.^13^ According to a review of the Surveillance, Epidemiology, and End Results (SEER) database of elderly breast cancer patients, women who received adjuvant anthracyclines had significantly higher rates of heart failure compared with those who received nonanthracycline adjuvant regimens.^14^ Despite extensive studies, the mechanism of anthracycline‐induced cardiac dysfunction is still unclear. Although many experts believe it is multifactorial, the following are the two main accepted hypotheses: (i) Inhibition of DNA topoisomerase IIβ, in cardiomyocytes, anthracyclines inhibit mitochondrial biogenesis while activating death pathways by binding to topoisomerase IIβ. (ii) Oxidative damage and free radical generation, myocytes are irreversibly damaged by anthracyclines because they generate reactive oxygen species (ROS) and free radicals, which cause lipid, nucleic acid, and protein oxidation, and tissue fibrosis.^15^

The majority of our study population were breast cancer patients. In addition to anthracyclines, trastuzumab, a monoclonal antibody against the HER2 receptor, also plays a crucial role in their treatment regimen. We performed a subgroup analysis of the incidence of chemotherapy‐related cardiac dysfunction according to breast cancer treatment (anthracyclines, trastuzumab, or anthracyclines plus trastuzumab). The cardiac dysfunction incidence rate was highest for anthracyclines plus trastuzumab, followed by trastuzumab, and lowest in anthracyclines. Although the difference was not statistically significant, there was a trend of increasing cardiac dysfunction risk associated with anthracyclines plus trastuzumab. The additive cardiotoxic effect of anthracyclines and trastuzumab may be due to the inhibition of HER‐2 signaling, decreased survival pathways, and increased oxidative and nitrative stress. Consequently, intracellular ROS accumulate, causing cardiac dysfunction and cardiomyocyte apoptosis. Besides, trastuzumab interferes with NRG/ErbB signaling, preventing its protective effects from anthracycline‐induced apoptosis, and causing calcium dysregulation and mitochondrial dysfunction.^16^

Also, a higher incidence of chemotherapy‐related cardiac dysfunction in cancer patients aged 50 years and over was found, which was similar to a previous study.^17^ There is no doubt that age is a risk factor for cardiac dysfunction. It is quite likely that among older patients, hypertension, diabetes, and cardiovascular illness were more prevalent, which are also the risk factors for cardiac dysfunction.^18^ In addition, there was a clear majority of female patients in the present study. The estrogen in women has stopped protecting them as they have aged, which is also a possible cause.^19^

Furthermore, the risk of chemotherapy‐related cardiac dysfunction in cancer patients varied during the follow‐up period of the disease. After chemotherapy, the risk of cardiac dysfunction increased initially (6 months), then declined gradually and became insignificant. Sandamali et al. reported that 6 months after chemotherapy, a total of 65 patients out of 196 (33.16%) developed cardiac dysfunction, which was relatively higher in these patients.^20^ Nevertheless, another study found that the cumulative incidence of cardiac dysfunction was 17.5% at 5 years.^21^ The reason for this result may be due to the fact that chemotherapy treatment is most intense in the early stages and then attenuates over time. Besides, patients undergo adjuvant radiation therapy early in treatment, which also is prone to cardiac dysfunction. Alternatively, cancer patients probably received more detection in the early period, resulting in a faster discovery of chemotherapy‐related cardiac dysfunction.

It is important to note some of the implications of this meta‐analysis. Patients with specific cancer types, and at the start of their cancer chemotherapy journey, may be motivated by the results to attend screening examinations of cardiac dysfunction. To mitigate the risk of chemotherapy‐related cardiac dysfunction in cancer patients, clinicians should meticulously evaluate the optimal chemotherapy treatment strategies while incorporating cardioprotective measures. This necessitates the collaboration of multidisciplinary teams.

There are several strengths of this meta‐analysis, including the strict criteria for inclusion, the large number of patients, the study participants' diversity, and the relationship between follow‐up duration and risk of chemotherapy‐related cardiac dysfunction. Besides, for the study precision and reliability, studies with a small sample size were not included in this study.

However, there exist a couple of limitations in the research. First, the study has the limitations of being a retrospective analysis. Second, this meta‐analysis, like other meta‐analyses, had a high statistical heterogeneity and remained in the subgroup analysis, which is difficult to avoid in a meta‐analysis of observational research. The study designs, data materials, analytical approaches, durations, and quality varied between studies, which may have contributed to the heterogeneity of the studies. Third, most of the included patients were breast cancer patients, which could have influenced the overall incidence. Similarly, the majority of breast cancer patients were female, so more studies with a high female proportion were included. We conducted stratification analyses according to sex distribution (female proportion), which showed no appreciable difference between the results for both sexes. However, the analysis was limited to only six studies with a high male predominance, resulting in highly uncertain estimates for males. Also, the maximum follow‐up time of the included studies was 9.23 years. We failed to explore the cardiac dysfunction occurring after 9.23 years days or more. Besides, cardioprotective therapy may prevent or mitigate chemotherapy‐related cardiac dysfunction, but specific data on evaluating if a cardioprotective treatment is started and in which patients were missing, which may have an impact on results. Thus, it can not further explain the decline in the incidence of the disease in the following years after treatment and during of monitoring. In addition, this study relies on published data that may have been biased by publication. Finally, there was no comprehensive data available on individual patients in the studies, making it difficult to identify the independent associations between individual variables and chemotherapy‐related cardiac dysfunction. Thus, to better understand this risk, larger population‐based longitudinal cohorts are needed. It is warranted to further investigate the underlying mechanisms and evaluate potential risk factors to develop better monitoring and treatment strategies against chemotherapy‐related cardiac dysfunction.

## 5 CONCLUSION

In conclusion, our meta‐analysis unveiled a definitive overall incidence rate of chemotherapy‐related cardiac dysfunction in cancer patients. In addition, it was found that the risk of developing this condition escalates within the initial 6 months postchemotherapy, subsequently tapering off to become statistically insignificant after a duration of 6 years. Results from our study indicate a need for early screening and continuous monitoring of chemotherapy‐related cardiac dysfunction in cancer patients.

## CONFLICT OF INTEREST STATEMENT

The authors declare no conflict of interest.

## Supporting information

Supporting information.

Supporting information.

## Data Availability

The data that support the findings of this study are available from the corresponding author upon reasonable request. The original contributions presented in the study are included in the article/Supplementary Material, further inquiries can be directed to the corresponding authors.
