# Supplementary material for: Incidence of chemotherapy‐related cardiac dysfunction in cancer patients
Source: Clin Cardiol. 2024 Apr 18;47(4):e24269. doi: 10.1002/clc.24269 (PMC11024952; doi:10.1002/clc.24269)

**Supplement Figure S1. Funnel plot.**


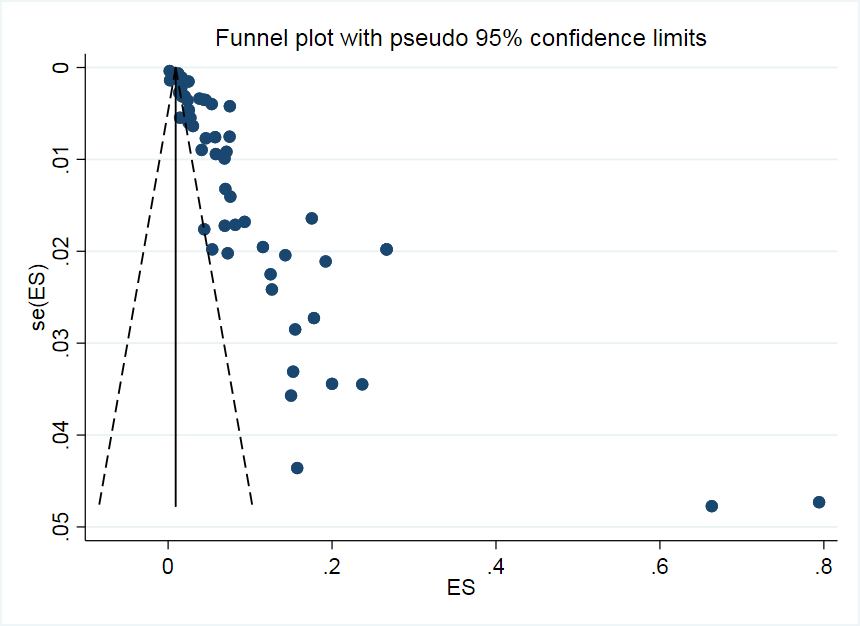


**Supplement Figure S2. Forest plot showing the incidence of chemotherapy-related** **cardiac dysfunction according to study type.**


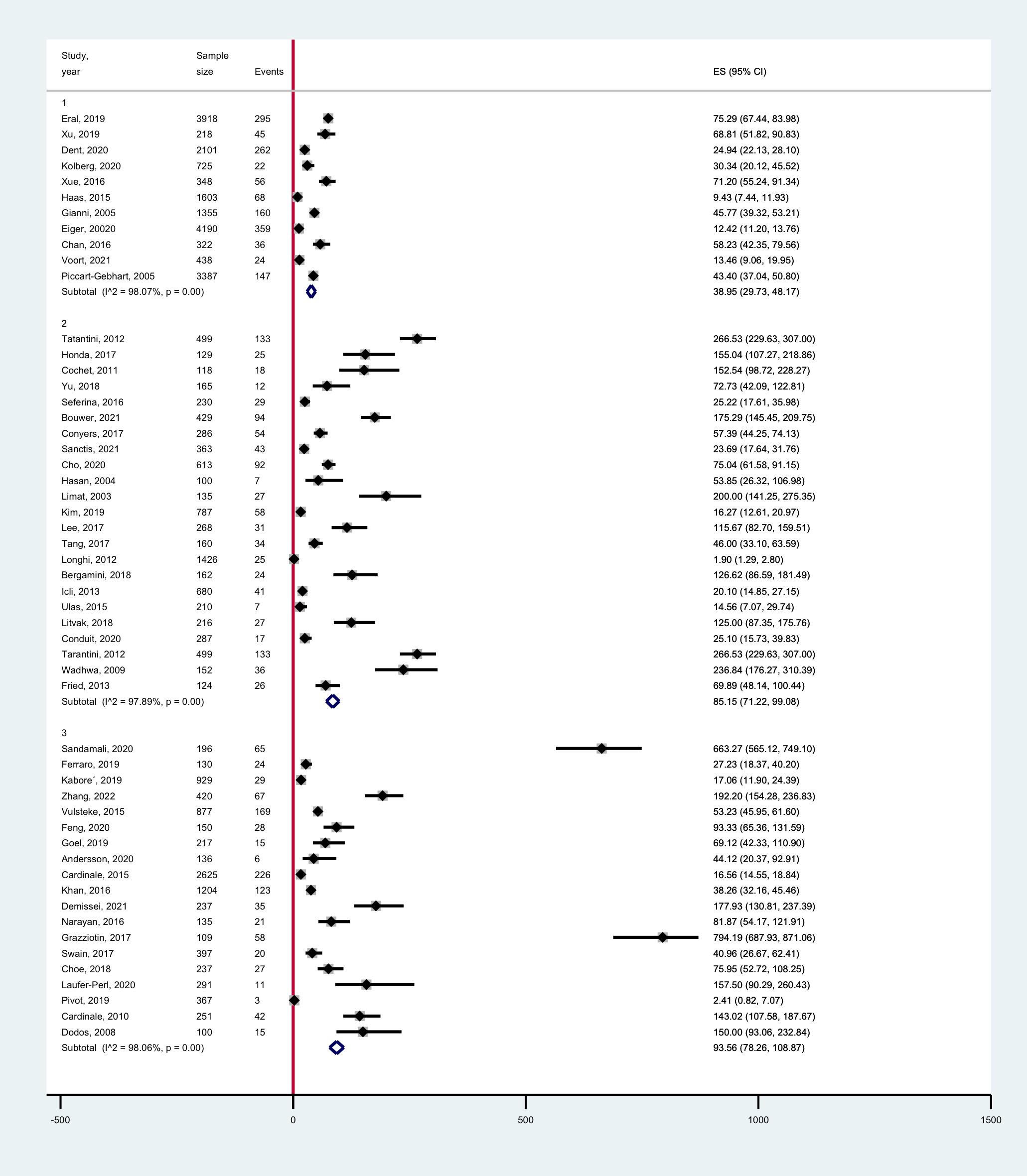


**Supplement Figure S3. Forest plot showing the incidence of chemotherapy-related cardiac dysfunction according to sample size.**


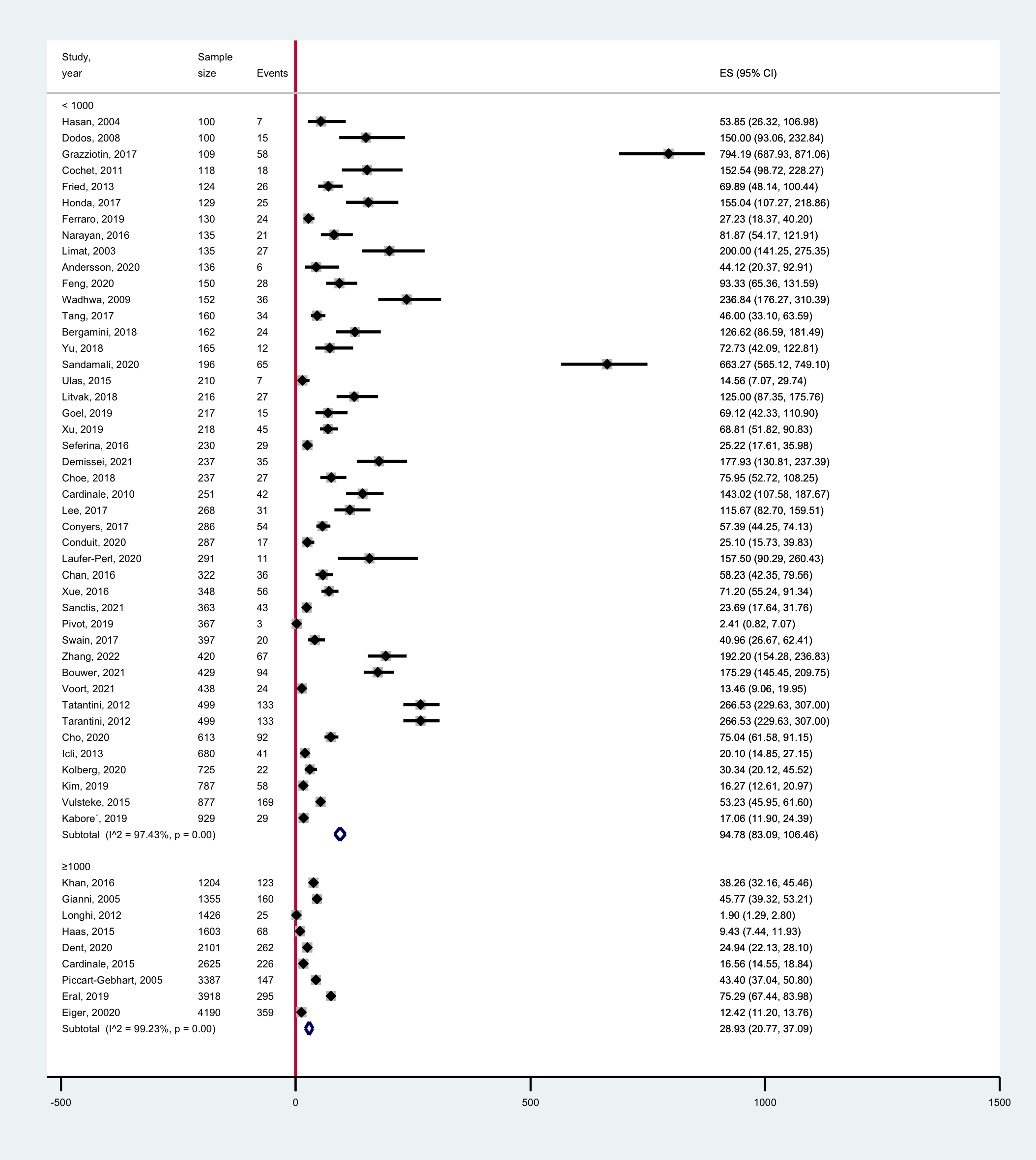


**Supplement Figure S4. Forest plot showing the incidence of chemotherapy-related cardiac dysfunction according to age.**


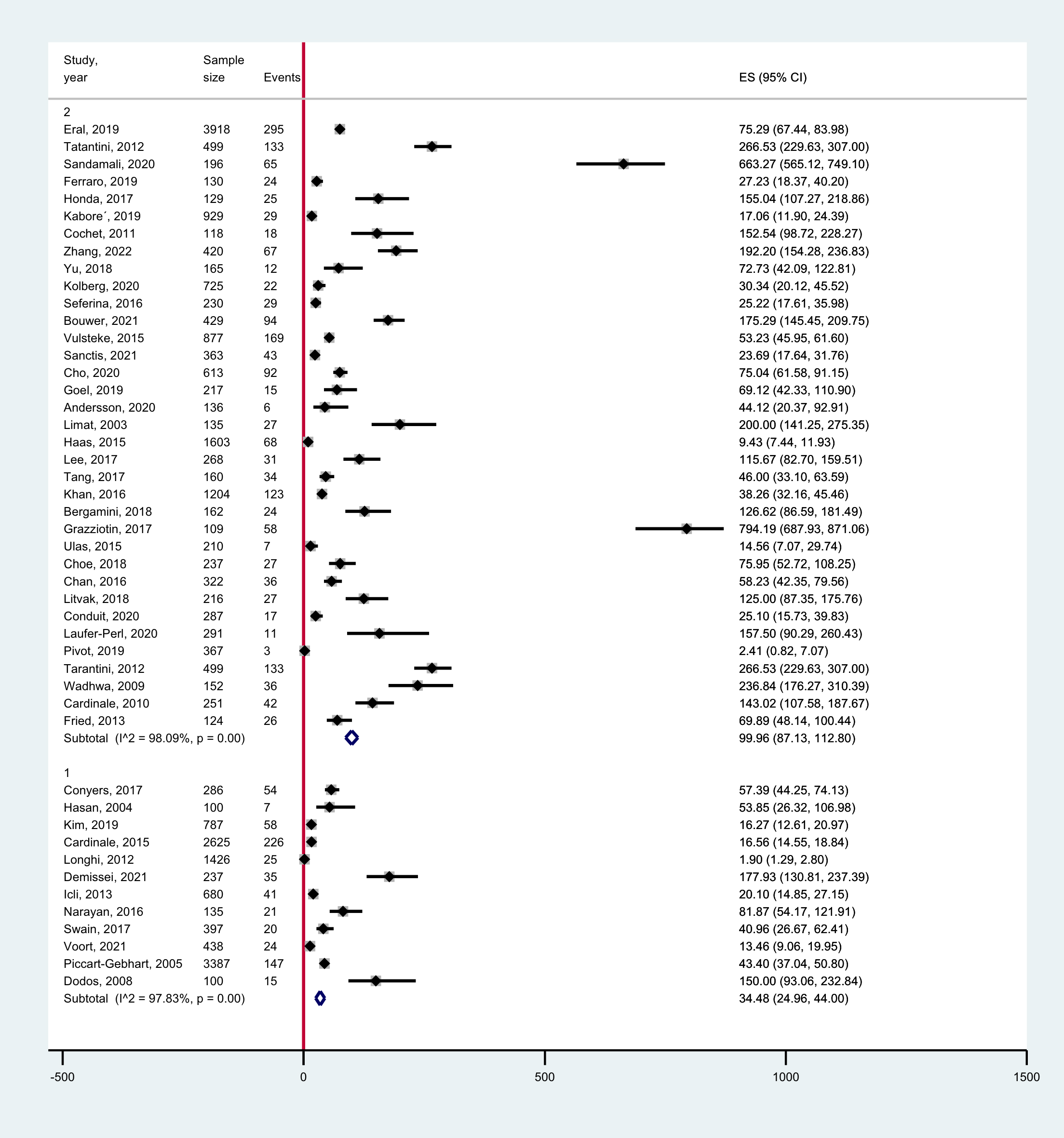


**Supplement Figure S5. Forest plot showing the incidence of chemotherapy-related cardiac dysfunction according to female proportion.**


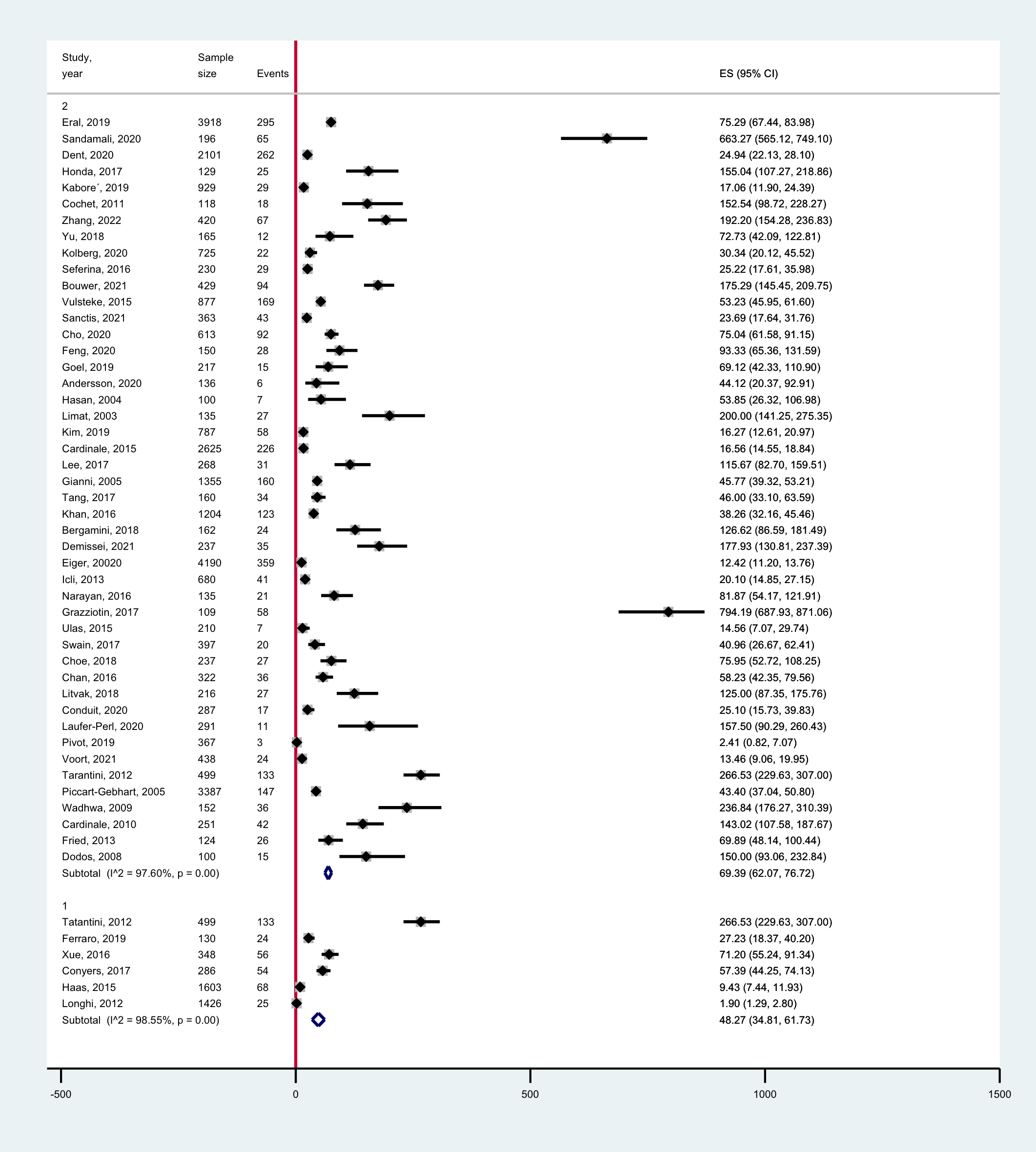


**Supplement Figure S6. Forest plot showing the incidence of chemotherapy-related cardiac dysfunction according to location.**


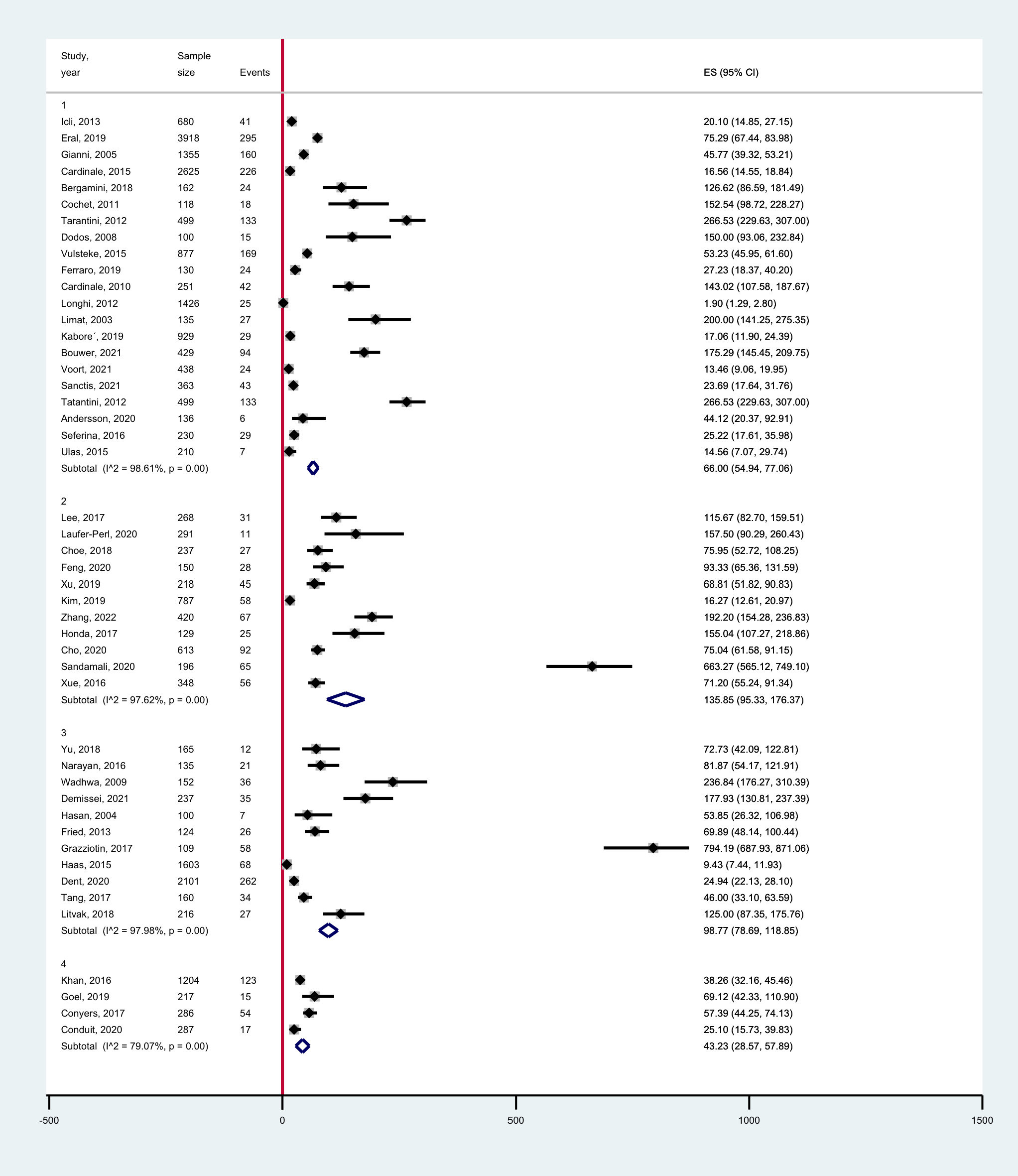


**Supplement Figure S7. Forest plot showing the incidence of chemotherapy-related cardiac dysfunction according to cancer type.**


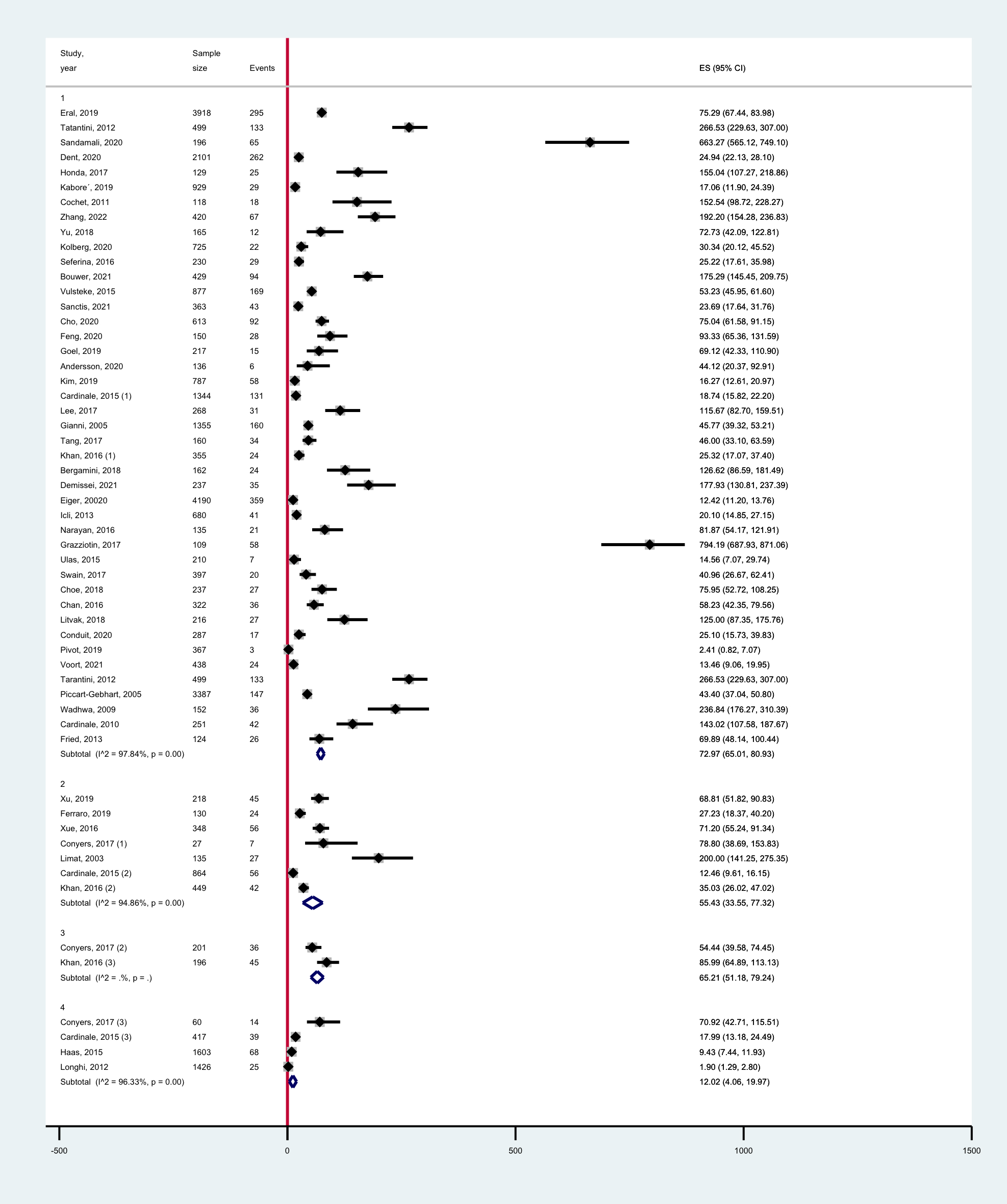


**Supplement Figure S8. Forest plot showing the incidence of chemotherapy-related cardiac dysfunction according to publication year.**


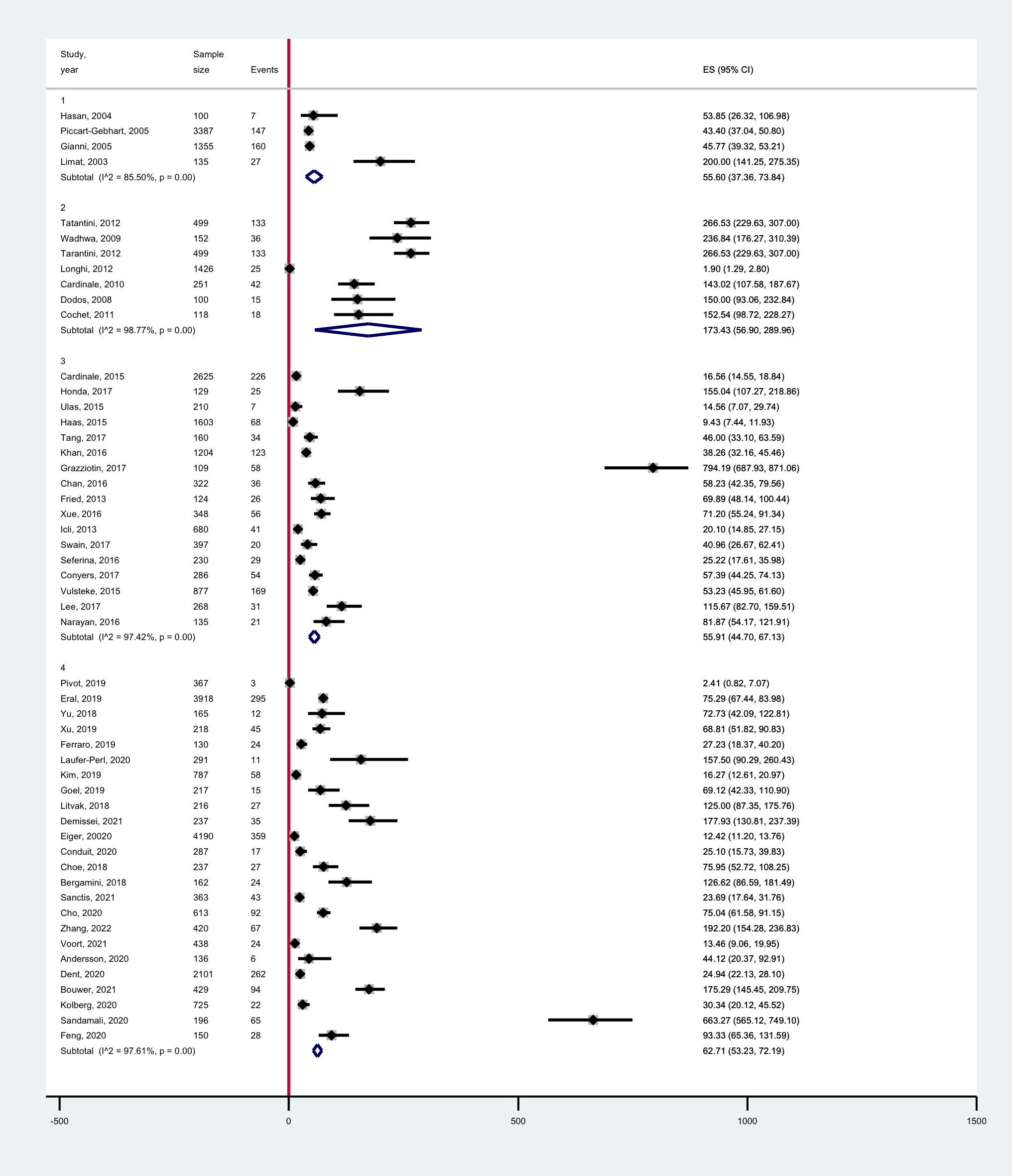


**Supplement Figure S9. Forest plot showing the incidence of chemotherapy-related cardiac dysfunction according to criterion.**


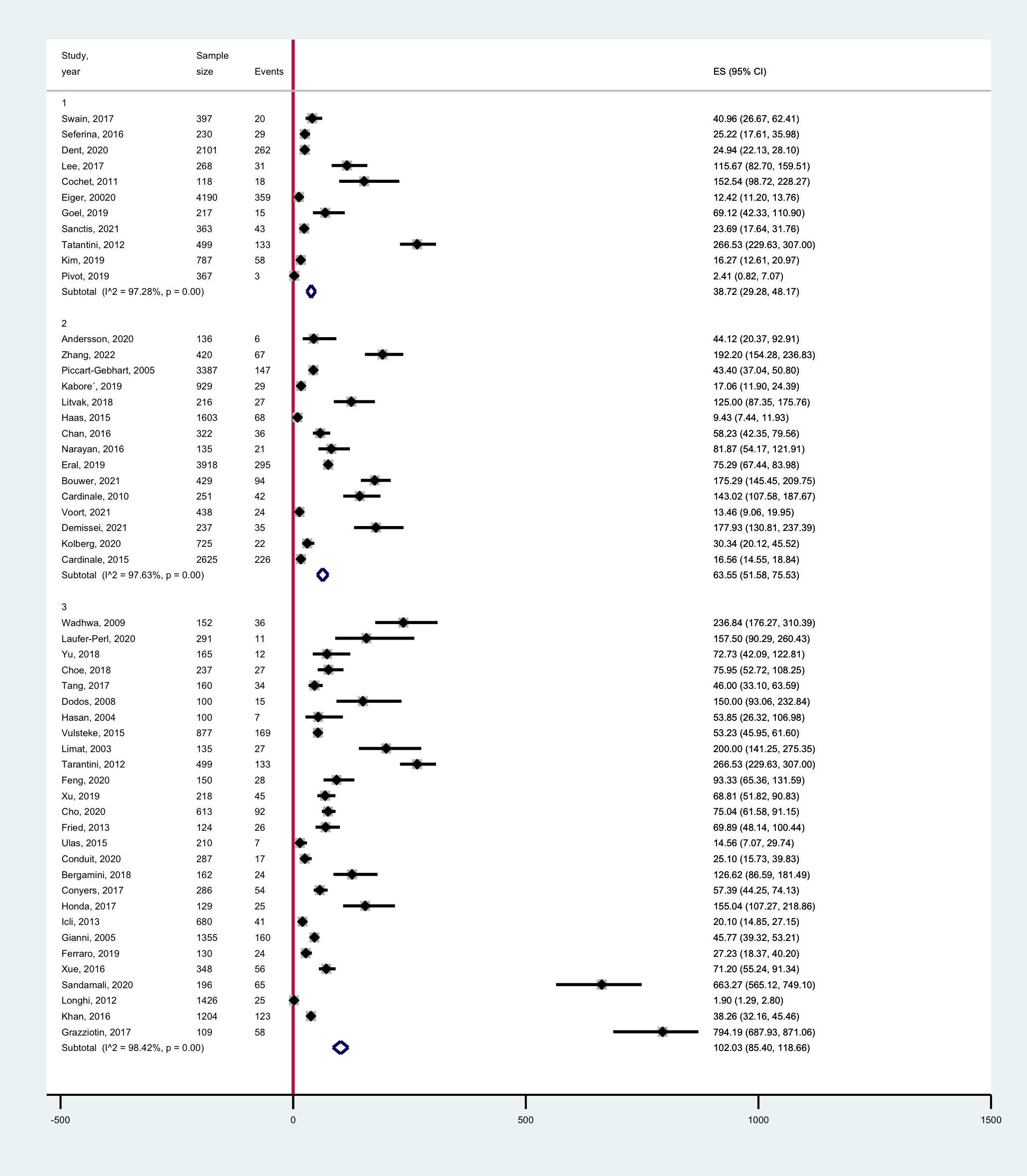


**Supplement Figure S10. Forest plot showing the incidence of chemotherapy-related cardiac dysfunction according to breast cancer treatment.**


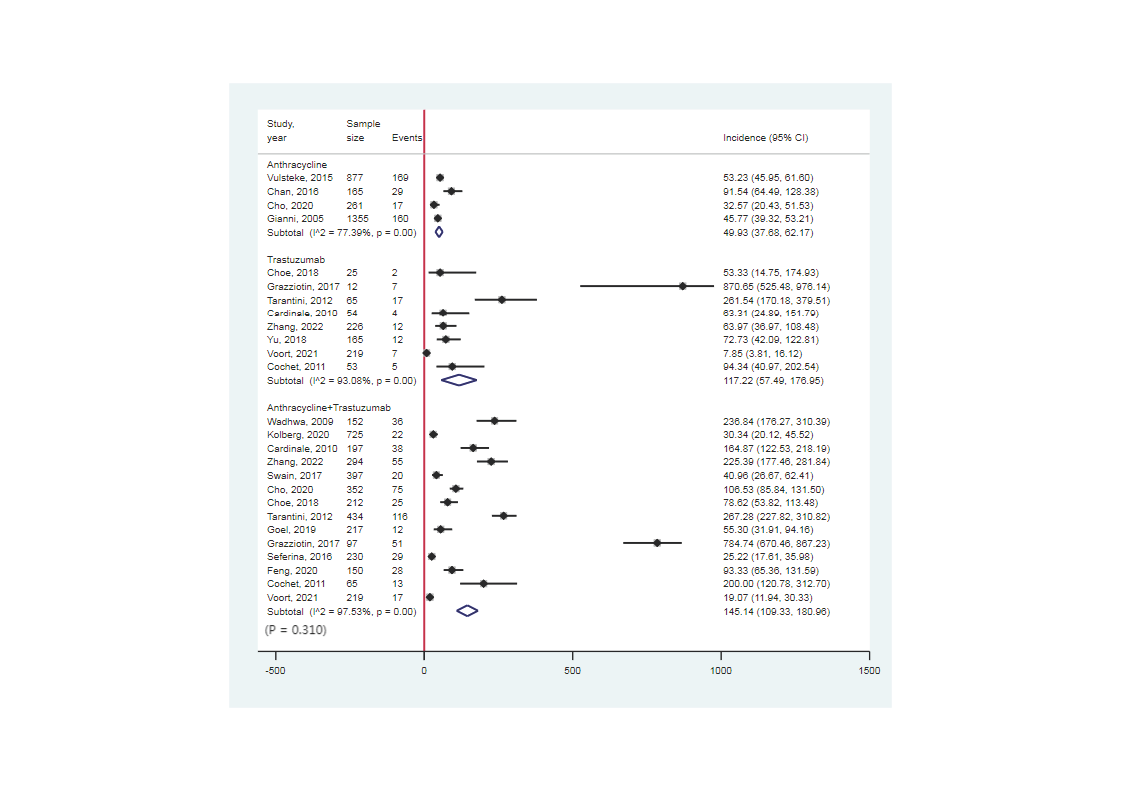

Supplement: Supplementary file 1 — Supporting information. [file CLC-47-e24269-s002.docx]
