# Supplementary material for: Incidence of chemotherapy‐related cardiac dysfunction in cancer patients
Source: Clin Cardiol. 2024 Apr 18;47(4):e24269. doi: 10.1002/clc.24269 (PMC11024952; doi:10.1002/clc.24269)
Supplement: Supplementary file 2 — Supporting information. [file CLC-47-e24269-s001.docx]

| **Table S1. Summary of Available Studies Included in the Present Meta-analysis.** | | | | | | | | | | | | | |
| --- | --- | --- | --- | --- | --- | --- | --- | --- | --- | --- | --- | --- | --- |
| **Study, year** | **Year** | **Region** | **Data Sources** | **Design** | **Follow-up (years)** | **Sample size** | **Age (year)** | **Female (%)** | **Cancer type** | **Treatment** | **Events** | **Criterion** | **Tool** |
| Eral, 2019(1) | 2019 | UK | 152 centres in the UK | RCT | 1 | 3918 | 56 | 100 | HER2-positive early breast cancer | Anthracycline-based, Taxane-based, Anthracycline-based and taxane-based, No taxane and no anthracycline + Trastuzumab | 295 | LVEF decrease ≥10% from baseline to <50% | Echocardiography |
| Tatantini, 2012(2) | 2012 | Intaly | 10 Italian hospitals and cancer institutes | Retrospective | 1 | 499 | 55 | 20 | HER2-positive early breast cancer | Anthracycline-based, Taxane-based, Cyclophosphamide, 5-Fluorouracil + Trastuzumab | 133 | asymptomatic decline in LVEF >10 % from baseline evaluation; asymptomatic decline in LVEF below 50%; heart failure responsive to treatment | Echocardiography |
| Xu, 2019(3) | 2019 | China | 20 centres of the Multicenter Hematology–Oncology Programs Evaluation System in China. | RCT | 3 | 218 | - | - | Diffuse large B-cell lymphoma | (Rituximab, cyclophosphamide, doxorubicin 50mg/m2, vincristine,and prednisone) or (rituximab, cyclophosphamide, epirubicin 70 mg/m², vincristine, and prednisone) | 45 | LVEF decrease ≥10% | Echocardiography |
| Sandamali, 2020(4) | 2020 | Sri Lanka | Teaching Hospital, Karapitiya | Prospective | 0.5 | 196 | 53.6 | 100 | Breast cancer | Anthracycline and cyclophosphamide | 65 | LVEF decrease > 10% | Echocardiography |
| Ferraro, 2019(5) | 2019 | Spain | Hospital del Mar | Prospective | 6.78 | 130 | 68 | 49 | Diffuse large B-cell lymphoma | First‐line with anthracycline‐containing immunochemotherapy | 24 | LVEF <55%, a 15% decline or more of baseline LVEF if baseline LVEF was <55% or clinical evidence of heart failure. | Echocardiography |
| Dent, 2020(6) | 2020 | Canada, USA | multicentre | RCT | 5 | 2101 | - | 100 | Early breast cancer | Cyclophosphamide (75 mg/m2) orally for 14 days, epirubicin (60 mg/m2) and fluorouracil, IV days one and eight (CEF) for six cycles; dose-dense epirubicin (120 mg/m2) and cyclophosphamide, IV every 2 weeks for six cycles with concurrent G-CSF then paclitaxel every 2 weeks for four cycles (ddEC/T); doxorubicin (60 mg/m2) and cyclophosphamide (600 mg/m2) every 3 weeks for four cycles then four cycles q3 weekly paclitaxel (175 mg/m2) (AC/T) | 262 | LVEF decrease ≥ 10% and an absolute value of less than 50% or heart failure | Echocardiography or MUGA |
| Honda, 2017(7) | 2017 | Janpa | Nagoya University Hospital | Retrospective | 1.25 | 129 | 54 | 100 | HER2-positive early breast cancer | Trastuzumab at a dose of 8 mg/kg in the first cycle and 6 mg/kg in the subsequent cycles, given every 3 weeks. | 25 | LVEF <50% or LVEF decrease ≥ 10%. | Echocardiography |
| Kabore´, 2019(8) | 2019 | France | 2012–2014 in the French national multicenter prospective CANTO (CANcer TOxicities) study of 26 French cancer centers | Prospective | 1.83 | 929 | 52.4 | 100 | Non-metastatic invasive breast cancer | The anthracycline regimens administered to patients included epirubicin (86%) and doxorubicin (7%). Trastuzumab was administered alone to 67 patients (7%) and combined with standard chemotherapy including anthracycline for 338 patients (36%). | 29 | LVEF decrease >10% from baseline to <50% | Echocardiography or MUGA |
| Cochet, 2011(9) | 2011 | France | Centre Georges-Franc¸ois Leclerc | Retrospective | 1 | 118 | 59 | 100 | HER2-positive early breast cancer | Trastuzumab was initially administered intravenously at a loading dose of 8 mg/kg, followed by maintenance doses of 6 mg/kg every 3 weeks for 1 year | 18 | Asymptomatic decline in LVEF ≥10% from baseline evaluation; heart failure responsive to treatment | Radionuclide angiocardiography |
| Zhang, 2022(10) | 2022 | China | Tianjin Medical University Cancer Hospital | Prospective | 0.83 | 420 | 52 | 100 | HER2-positive early breast cancer | Trastuzumab,trastuzumab and pertuzumab | 67 | LVEF decrease >10% from baseline to <50% | Echocardiography |
| Yu, 2018(11) | 2018 | USA | - | Retrospective | 1 | 165 | 59 | 100 | HER2-positive early breast cancer | Non-anthracycline trastuzumab-based therapy | 12 | Asymptomatic LVEF decrease ≥ 10% from baseline to below 55% or ≥ 16% (and above the lower limit of normal); HF | Echocardiography or MUGA or CMR |
| Kolberg, 2020(12) | 2020 | Multicentre | LILAC study | RCT | 1 | 725 | 52.8 | 100 | HER2-positive early breast cancer | epirubicin and cyclophosphamide + Trastuzumab | 22 | LVEF decrease >10% from baseline to <50% | Echocardiography |
| Seferina, 2016(13) | 2016 | Netherlands | Five hospitals in Southeast Netherlands | Retrospective | 5 | 230 | 51.2 | 100 | HER2-positive early breast cancer | Trastuzumab following initial adjuvant chemotherapy | 29 | LVEF decrease >10% from baseline to <50%; HF | Echocardiography |
| Xue, 2016(14) | 2016 | China | Fudan University Shanghai Cancer Center, Shanghai | RCT | 2.26 | 348 | - | 47.4 | Lymphoma | 6-8 cycles of cyclophosphamide, vincristine and prednisone (CEpOP) +/- rituximab (R) with either epirubicin (CEpOP) or doxorubicin (CHOP) | 56 | LVEF <50%; LVEF decrease ≥ 10%; HF | MUGA |
| Bouwer, 2021(15) | 2021 | Netherlands | Netherlands Cancer Registry | Retrospective | 1.25 | 429 | 54 | 100 | HER2-positive metastatic breast cancer | Trastuzumab-based treatment | 94 | LVEF decrease >10% from baseline to <50% | MUGA |
| Conyers, 2017(16) | 2017 | Australia | Haematology-Oncology database at the Royal Children’s Hospital, Melbourne | Retrospective | 3.29 | 286 | 8.8 | 45.8 | Leukaemia, lymphoma, osteosarcoma, rhabdomyosarcoma, Ewing sarcoma, desmoplastic small round cell tumour, neuroblastoma, nephroblastoma,other | Anthracyclines | 54 | Fractional shortening(FS) <24%; FS 24–28%; LVEF decrease >10% | Echocardiography |
| Vulsteke, 2015(17) | 2015 | Belgium | Leuven Multidisciplinary Breast Cancer Center | Prospective | 3.62 | 877 | 50.3 | 100 | Early breast cancer | (neo) adjuvant 5-fluorouracil, epirubicin and cyclophosphamide | 169 | Asymptomatic LVEF decrease > 10%; HF | Echocardiography or MUGA |
| Sanctis, 2021(18) | 2021 | Italy | Humanitas Cancer Center | Retrospective | 5 | 363 | 55 | 99.7 | HER2-positive early breast cancer | Trastuzumab-based treatment | 43 | Asymptomatic LVEF decrease ≥ 10% from baseline to below 50%; HF | Echocardiography |
| Cho, 2020(19) | 2020 | Korea | National Cancer Center | Retrospective | 2 | 613 | 54.1 | 100 | Breast cancer | Chemotherapy regimens including doxorubicin were as follows: (1) doxorubicin and cyclophosphamide (AC), (2) 5-fluorouracil, doxorubicin, and cyclophosphamide (FAC), (3) doxorubicin and docetaxel or paclitaxel, (4) AC or FAC followed by paclitaxel, and (5) AC or FAC followed by paclitaxel, then trastuzumab (concomitant trastuzumab) | 92 | Echocardiography: LVEF decrease > 10% from baseline to < 55% or > 15% with LVEF > 55%; MUGA: LVEF decrease> 10% points from baseline and LVEF < 50% or > 15% with LVEF > 50%. | Echocardiography or MUGA |
| Feng, 2020(20) | 2020 | China | Guangxi Medical University Cancer Hospital | Prospective | 2 | 150 | - | 100 | HER2-positive breast cancer | (a) adjuvant chemotherapy based on anthracyclines doxorubicin (ADM) or epirubicin (EPI) combined with taxanes was completed, and continuously receive trastuzumab | 28 | LVEF <50%; LVEF decrease ≥ 10% | Echocardiography |
| Goel, 2019(21) | 2019 | Australia | 17 Australian institutions | Prospective | 1 | 217 | 52 | 100 | HER2-positive breast cancer | Conventional adjuvant anthracycline-based chemotherapy followed by taxane chemotherapy given with trastuzumab, followed by trastuzumab alone to complete a total of 52 weeks | 15 | LVEF decrease ≥ 10% from baseline to below 50%; HF | Echocardiography or MUGA |
| Andersson, 2020(22) | 2020 | Sweden | Department of Oncology, Sahlgrenska University Hospital, Gothenburg | Prospective | 1 | 136 | 56.8 | 99.3 | HER2-positive breast cancer | The majority of patients were treated with trastuzumab (HerceptinVR ) subcutaneously (600 mg) or intravenously (6 mg/kg, start dose 8 mg/kg) in the adjuvant setting (85%), while a small number of patients were treated in the neoadjuvant or the palliative settings | 6 | LVEF decrease ≥ 10% from baseline to below 50% | Echocardiography |
| Hasan, 2004(23) | 2004 | USA | Howard University Hospital cancer registry | Retrospective | 1.3 | 100 | 46 | 65 | - | Doxorubicin-based combination chemotherapy | 7 | LVEF <45%, heart failure | Echocardiography |
| Limat, 2003(24) | 2003 | France | Besançon University Hospital | Retrospective | 1 | 135 | 59 | 57 | non-Hodkin's lymphoma | CHOP (cyclophosphamide, doxorubicin, vincristin, prednisone) regimen | 27 | LVEF <50%; LVEF decrease ≥ 15%; HF | Echocardiography |
| Kim, 2019(25) | 2019 | Korea | Samsung Medical Center | Retrospective | 4.53 | 787 | 49.9 | 100 | HER2-positive breast cancer | Adjuvant therapy with trastuzumab | 58 | LVEF decrease ≥10% from baseline to <50%; HF | Echocardiography |
| Cardinale, 2015(26) | 2015 | Milan | European Institute of Oncology | Prospective | 5.2 | 2625 | 49.2 | 74.2 | Breast cancer, Hodgkin disease, Non-Hodgkin lymphoma, Myeloma, Ovarian, Other hemato-oncologic diseases, Other solid tumors | Anthracycline containing chemotherapy | 226 | LVEF decrease >10% from baseline to <50% | Echocardiography |
| Haas, 2015(27) | 2015 | USA | The E2805 trial | RCT | 4.5 | 1603 | 56 | 30.6 | Renal cell carcinoma | Nine 6-week cycles of either sunitinib 50 mg daily for 28 of 42 days per cycle, sorafenib 400 mg twice daily, or placebo | 68 | LVEF decrease ≥10% from baseline to <50% | MUGA |
| Lee, 2017(28) | 2017 | Korea | National Cancer Center | Retrospective | 1 | 268 | 50 | 100 | HER2-positive breast cancer | Trastuzumab-based treatment | 31 | LVEF decrease ≥10% from baseline to <50%; HF | Echocardiography |
| Gianni, 2005(29) | 2005 | Europe | The European CooperativeTrial in Operable breast cancer (ECTO) | RCT | 2.58 | 1355 | - | 100 | Breast cancer | Patients in arm A first received surgery followed by sequential single-agent doxorubicin and CMF; patients in arm B first underwent surgery followed by sequential doxorubicin/paclitaxel and CMF; patients in arm C received sequential doxorubicin/paclitaxel and CMF followed by surgery | 160 | LVEF <50% or LVEF decrease > 20%. | Echocardiography |
| Tang, 2017(30) | 2017 | Canada | St. Michael’s Hospital, Toronto | Retrospective | 4.62 | 160 | 52.4 | 100 | HER2-positive breast cancer | Adjuvant chemotherapy with trastuzumab | 34 | Asymptomatic LVEF decrease ≥ 10% or ≤ 50%; HF | Echocardiography or MUGA or CMR |
| Khan, 2016(31) | 2016 | Australia | Hunter New England Local Health Distric | Prospective | 2.67 | 1204 | 58 | 64 | Breast cancer,lymphoma, leukaemia | Anthracycline-based chemotherapy | 123 | LVEF <55% and LVEF decrease ≥ 10%. | Echocardiography |
| Longhi, 2012(32) | 2012 | Italy | Institute (Rizzoli Orthopedic Institute [IOR]) in Bologna, Italy and in other Italian Sarcoma Group (ISG) centers | Retrospective | 9.23 | 1426 | 15.4 | 40.7 | Osteosarcoma, Ewing sarcoma | Anthracycline-based chemotherapy | 25 | LVEF <50%. | Echocardiography |
| Bergamini, 2018(33) | 2018 | Italy | University of Verona | Retrospective | 1.17 | 162 | 58 | 100 | HER2-positive early breast cancer | Trastuzumab-based treatment | 24 | LVEF <50%; LVEF decrease ≥ 10%; HF | Echocardiography |
| Demissei, 2021(34) | 2021 | USA | Cardiotoxicity of Cancer Therapy (CCT) study | Prospective | 0.83 | 237 | 48 | 100 | Breast cancer | Either doxorubicin (240mg/m2 divided into four cycles of 60mg/m2 each) and cyclophosphamide followed by a taxane-containing regimen (Doxorubicin) or doxorubicin (240mg/m2 divided into four cycles of 60mg/m2 per cycle) and cyclophosphamide followed by a taxane and trastuzumab (Doxorubicin + Trastuzumab). | 35 | LVEF decrease ≥10% from baseline to <50% | Echocardiography |
| Eiger, 2020(35) | 2020 | Multi-centre | The Adjuvant Lapatinib and/or Trastuzumab Treatment Optimization ALTTO | RCT | 6.9 | 4190 | - | 100 | HER2-positive early breast cancer | Anthracycline, taxanes + Trastuzumab and/or Lapatinib | 359 | LVEF decrease ≥10% from baseline to <50%; HF | Echocardiography or MUGA |
| Icli, 2013(36) | 2013 | Turkey | Turkish Ministry of Health | Retrospective | 3 | 680 | 47.7 | 100 | HER2-positive early breast cancer | Trastuzumab-based treatment | 41 | LVEF decrease ≥ 10% | Echocardiography |
| Narayan, 2016(37) | 2016 | USA | Rena Rowan Breast Cancer Center of the Abramson Cancer Center at the University of Pennsylvania | Prospective | 1.9 | 135 | 48 | 100 | Breast cancer | 1) doxorubicin (240 mg/m2) and cyclophosphamide, followed by paclitaxel; 2) doxorubicin (240 mg/m2) and cyclophosphamide followed by paclitaxel and trastuzumab; or 3) cyclophosphamide or carboplatin with docetaxel and trastuzumab | 21 | LVEF decrease ≥10% from baseline to <50% | Echocardiography |
| Grazziotin, 2017(38) | 2017 | Brazil | Hospital Nossa Senhora da Conceic¸a˜o, Hospital Feˆmina and Hospital de Clı´nicas de Porto Alegre | Prospective | 0.67 | 109 | 53.9 | 100 | HER2-positive breast cancer | Trastuzumab-based treatment | 58 | LVEF <50%; LVEF decrease ≥ 10%; HF | Echocardiography |
| Ulas, 2015(39) | 2015 | Turkey | Bursa Oncology hospital and Balıkesir public hospital | Retrospective | 2.29 | 210 | 51.8 | 100 | HER2-positive early breast cancer | Adjuvant trastuzumab based treatment | 7 | LVEF <50% or LVEF decrease > 10%. | Echocardiography |
| Swain, 2017(40) | 2017 | Multicentre | BERENICE | Prospective | 1.23 | 397 | 49 | 100 | HER2-positive breast cancer | Pertuzumab, trastuzumab, and standard anthracycline- and taxane-based chemotherapy | 20 | LVEF decrease ≥10% from baseline to <50%; HF | Echocardiography or MUGA |
| Choe, 2018(41) | 2018 | Korea | Pusan National University Hospital | Prospective | 1.5 | 237 | 53.9 | 100 | HER2-positive breast cancer | Trastuzumab-based treatment | 27 | LVEF <55%, either as symptomatic decrease of ≥5% or asymptomatic decrease of ≥10%. | Echocardiography |
| Chan, 2016(42) | 2016 | Multicentre | 41 centers worldwide | RCT | 1.92 | 322 | 52 | 100 | Metastatic breast cancer | Docetaxel, doxorubicin | 36 | LVEF decrease ≥10% from baseline to <50% | Echocardiography or MUGA |
| Litvak, 2018(43) | 2018 | USA | Sidney Kimmel Comprehensive Cancer Center at Johns Hopkins | Retrospective | 1 | 216 | 53 | 100 | HER2-positive early breast cancer | Neoadjuvant or adjuvant trastuzumab with or without pertuzumab | 27 | LVEF decrease ≥10% from baseline to <50% | Echocardiography or MUGA |
| Conduit, 2020(44) | 2020 | Australia | TABITHA registry | Retrospective | 2.36 | 287 | 57 | 99.7 | HER2-positive metastatic breast cancer | Dual HER2 blockade with trastuzumab and pertuzumab, trastuzumab monotherapy, T-DM1, pertuzumab monotherapy or lapatinib. | 17 | Asymptomatic LVEF decrease ≥ 10% from baseline to below 50% or symptomatic LVEF decrease ≥ 10% from baseline. | Echocardiography or MUGA |
| Laufer-Perl, 2020(45) | 2020 | Israel | Israel Cardio-Oncology Registry (ICOR) | Prospective | 0.24 | 291 | 62 | 70 | Breast, sarcoma, lung, gastrointestinal, genitourinary, hematologic, other types | Anthracycline-based chemotherapy | 11 | LVEF decrease >10% from baseline to <53% | Echocardiography |
| Pivot, 2019(46) | 2019 | Multicentre | The extension study (NCT02771795, EudraCT 2015-005663-17) | Prospective | 3.39 | 367 | 52.5 | 100 | HER2-positive breast cancer | Neoadjuvant treatment, surgery and adjuvant treatment + Trastuzumab biosimilar (SB3) or trastuzumab | 3 | LVEF decrease ≥10% from baseline to <50%; HF | Echocardiography or MUGA |
| Voort, 2021(47) | 2021 | Netherlands | TRAIN-2 | RCT | 4.07 | 438 | 48.5 | 100 | ERBB2-positive breast cancer | Patients in the nonanthracycline group received nine 3-week cycles of paclitaxel (80mg/m2 on days 1 and 8) and carboplatin (area under the concentration-time curve [AUC], 6mg/mL/min on day 1 or AUC, 3mg/mL/min on days 1 and 8). Patients in theanthracycline groupreceived3 cycles of fluorouracil (500mg/m2), epirubicin (90 mg/m2), and cyclophosphamide (500 mg/m2) intravenously for 3 weeks followed by 6 cycles of paclitaxel and carboplatin in the same schedule as in the nonanthracycline group. Trastuzumab (6mg/kg; loading dose 8mg/kg)andpertuzumab (420mg intravenously; loading dose 840mg) were concurrently administered with chemotherapy every 3 weeks to both groups | 24 | LVEF decrease ≥10% from baseline to <50% | Echocardiography |
| Tarantini, 2012(48) | 2012 | Italy | 10 Italian institutions | Retrospective | 1 | 499 | 55 | 100 | HER2-positive breast cancer | Trastuzumab-based treatment | 133 | LVEF decrease > 10%; HF | Echocardiography |
| Piccart-Gebhart, 2005(49) | 2005 | Multicentre | The Herceptin Adjuvant (HERA) (Breast Interna_x0002_tional Group [BIG] 01-01) Trial | RCT | 1 | 3387 | 49 | 100 | HER2-positive breast cancer | Trastuzumab-based treatment | 147 | LVEF decrease ≥10% from baseline to <50% | Echocardiography or MUGA |
| Wadhwa, 2009(50) | 2009 | Canada | University of Manitoba, Winnipeg, Manitoba | Retrospective | 1 | 152 | 52 | 100 | HER2-positive breast cancer | Trastuzumab in the adjuvant setting | 36 | Asymptomatic LVEF decrease ≥ 10% to below 55% or symptomatic LVEF decrease ≥ 5% to below 55%. | MUGA |
| Cardinale, 2010(51) | 2010 | Italy | European Institute of Oncol_x0002_ogy and Centro Cardiologico Monzino, Istituto Di Ricovero e Cura a Carattere Scientifico, Milan | Prospective | 1.17 | 251 | 50 | 100 | HER2-positive breast cancer | Trastuzumab-based treatment | 42 | LVEF decrease ≥10% from baseline to <50% | Echocardiography |
| Fried, 2013(52) | 2013 | USA | Division of Oncology, Rambam Health Care Campus | Retrospective | 3 | 124 | 51 | 100 | HER2-positive breast cancer | Trastuzumab-based treatment | 26 | LVEF <50% or LVEF decrease ≥ 10% or HF | Echocardiography |
| Dodos, 2008(53) | 2008 | Germany | University of Cologne | Prospective | 1 | 100 | 46.1 | 52 | Non-Hodgkin lymphoma, breast cancer, Hodgkins lymphoma, acute myeloic leukemia, multiple myeloma, acute lymphatic leukemia, lung cancer, sarcoma, chronic lymphatic leukemia, malignant histiocytoma, other malignancies | Anthracyclines chemotherapy | 15 | LVEF decrease ≥20% from baseline or LVEF decrease ≥10% from baseline to <55% or HF | Echocardiography |

**References:**

1. Hiller L, Vallier A, McAdam K, et al. 6 versus 12 months of adjuvant trastuzumab for HER2-positive early breast cancer (PERSEPHONE): 4-year disease-free survival results of a randomised phase 3 non-inferiority trial. The Lancet (British edition) 2019;393:2599-2612.

2. Tarantini L, Gori S, Faggiano P, et al. Adjuvant trastuzumab cardiotoxicity in patients over 60 years of age with early breast cancer: a multicenter cohort analysis. Annals of Oncology 2012;23:3058-3063.

3. Xu P, Fu D, Li J, et al. Anthracycline dose optimisation in patients with diffuse large B-cell lymphoma: a multicentre, phase 3, randomised, controlled trial. The Lancet. Haematology 2019;6:e328-e337.

4. Sandamali JAN, Hewawasam RP, Fernando MACS, et al. Anthracycline-Induced Cardiotoxicity in Breast Cancer Patients from Southern Sri Lanka: An Echocardiographic Analysis. BioMed Research International 2020;2020:1-8.

5. Ferraro MP, Gimeno Vazquez E, Subirana I, et al. Anthracycline‐induced cardiotoxicity in diffuse large B‐cell lymphoma: NT‐proBNP and cardiovascular score for risk stratification. European Journal of Haematology 2019.

6. Dent SF, Botros J, Rushton M, et al. Anthracycline-induced cardiotoxicity in patients with early-stage breast cancer: the Canadian Cancer Trials Group (CCTG) MA.21 experience. Breast Cancer Research and Treatment 2020;184:733-741.

7. Honda K, Takeshita K, Murotani K, et al. Assessment of left ventricular diastolic function during trastuzumab treatment in patients with HER2-positive breast cancer. Breast Cancer 2017;24:312-318.

8. Kaboré EG, Guenancia C, Vaz-Luis I, et al. Association of body mass index and cardiotoxicity related to anthracyclines and trastuzumab in early breast cancer: French CANTO cohort study. PLOS Medicine 2019;16:e1002989.

9. Cochet A, Quilichini G, Dygai-Cochet I, et al. Baseline diastolic dysfunction as a predictive factor of trastuzumab-mediated cardiotoxicity after adjuvant anthracycline therapy in breast cancer. Breast Cancer Research and Treatment 2011;130:845-854.

10. Zhang L, Wang Y, Meng W, Zhao W, Tong Z. Cardiac safety analysis of anti-HER2-targeted therapy in early breast cancer. Scientific Reports 2022;12.

11. Yu AF, Mukku RB, Verma S, et al. Cardiac safety of non-anthracycline trastuzumab-based therapy for HER2-positive breast cancer. Breast Cancer Research and Treatment 2017;166:241-247.

12. Kolberg H, Colleoni M, Demetriou GS, et al. Cardiac Safety of the Trastuzumab Biosimilar ABP 980 in Women with HER2-Positive Early Breast Cancer in the Randomized, Double-Blind, Active-Controlled LILAC Study. Drug Safety 2020;43:233-242.

13. Seferina SC, Boer M, Derksen MW, et al. Cardiotoxicity and Cardiac Monitoring During Adjuvant Trastuzumab in Daily Dutch Practice: A Study of the Southeast Netherlands Breast Cancer Consortium. The Oncologist 2016;21:555-562.

14. Xue K, Gu JJ, Zhang Q, et al. Cardiotoxicity as indicated by LVEF and troponin T sensitivity following two anthracycline-based regimens in lymphoma: Results from a randomized prospective clinical trial. Oncotarget 2016;7:32519-32531.

15. Bouwer NI, Steenbruggen TG, van Rosmalen J, et al. Cardiotoxicity during long-term trastuzumab use in patients with HER2-positive metastatic breast cancer: who needs cardiac monitoring? Breast Cancer Research and Treatment 2021;186:851-862.

16. Conyers R, Costello B, La Gerche A, et al. Chemotherapy‐related cardiotoxicity: are Australian practitioners missing the point? Internal Medicine Journal 2017;47:1166-1172.

17. Vulsteke C, Pfeil AM, Maggen C, et al. Clinical and genetic risk factors for epirubicin-induced cardiac toxicity in early breast cancer patients. Breast Cancer Research and Treatment 2015;152:67-76.

18. De Sanctis R, Giordano L, D Antonio F, et al. Clinical predictors of cardiac toxicity in HER2-positive early breast cancer patients treated with adjuvant s.c. versus i.v. trastuzumab. The Breast 2021;57:80-85.

19. Cho H, Lee S, Sim SH, et al. Cumulative incidence of chemotherapy-induced cardiotoxicity during a 2-year follow-up period in breast cancer patients. Breast Cancer Research and Treatment 2020;182:333-343.

20. Feng Y, Qin Z, Yang Z. Deceleration capacity of heart rate predicts trastuzumab‐related cardiotoxicity in patients with HER2‐positive breast cancer: A prospective observational study. Journal of Clinical Pharmacy and Therapeutics 2021;46:93-98.

21. Goel S, Liu J, Guo H, et al. Decline in Left Ventricular Ejection Fraction Following Anthracyclines Predicts Trastuzumab Cardiotoxicity. JACC: Heart Failure 2019;7:795-804.

22. Andersson AE, Linderholm B, Giglio D. Delta NT-proBNP predicts cardiotoxicity in HER2-positive breast cancer patients treated with trastuzumab. Acta Oncol 2021;60:475-481.

23. HASAN S, DINH K, LOMBARDO F, KARK J. Doxorubicin cardiotoxicity in African Americans. Journal of the National Medical Association 2004;96:196-199.

24. Limat S, Demesmay K, Voillat L, et al. Early cardiotoxicity of the CHOP regimen in aggressivenon-Hodgkin’s lymphoma. Annals of Oncology 2003;14:277-281.

25. Kim EK, Cho J, Kim J, et al. Early Decline in Left Ventricular Ejection Fraction Can Predict Trastuzumab-Related Cardiotoxicity in Patients with Breast Cancer: A Study Using 13 Years of Registry Data. Cancer Research and Treatment 2019;51:727-736.

26. Cardinale D, Colombo A, Bacchiani G, et al. Early Detection of Anthracycline Cardiotoxicity and Improvement With Heart Failure Therapy. Circulation 2015;131:1981-1988.

27. Haas NB, Manola J, Ky B, et al. Effects of Adjuvant Sorafenib and Sunitinib on Cardiac Function in Renal Cell Carcinoma Patients without Overt Metastases: Results from ASSURE, ECOG 2805. Clinical Cancer Research 2015;21:4048-4054.

28. Lee MH, Yee J, Kim YJ, et al. Factors for time to trastuzumab-induced cardiotoxicity in breast cancer patients. Medical Oncology 2017;34.

29. Gianni L, Baselga J, Eiermann W, et al. Feasibility and Tolerability of Sequential Doxorubicin/Paclitaxel Followed by Cyclophosphamide, Methotrexate, and Fluorouracil and Its Effects on Tumor Response as Preoperative Therapy. Clinical Cancer Research 2005;11:8715-8721.

30. Tang GH, Acuna SA, Sevick L, Yan AT, Brezden-Masley C. Incidence and identification of risk factors for trastuzumab-induced cardiotoxicity in breast cancer patients: an audit of a single “real-world” setting. Medical Oncology 2017;34.

31. Khan AA, Ashraf A, Singh R, et al. Incidence, time of occurrence and response to heart failure therapy in patients with anthracycline cardiotoxicity. Intern Med J 2017;47:104-109.

32. Longhi A, Ferrari S, Tamburini A, et al. Late effects of chemotherapy and radiotherapy in osteosarcoma and Ewing sarcoma patients. Cancer 2012;118:5050-5059.

33. Bergamini C, Dolci G, Rossi A, et al. Left atrial volume in patients with HER2-positive breast cancer: One step further to predict trastuzumab-related cardiotoxicity. Clinical Cardiology 2018;41:349-353.

34. Demissei BG, Fan Y, Qian Y, et al. Left ventricular segmental strain and the prediction of cancer therapy-related cardiac dysfunction. European Heart Journal - Cardiovascular Imaging 2021;22:418-426.

35. Eiger D, Pondé NF, Agbor-Tarh D, et al. Long-term cardiac outcomes of patients with HER2-positive breast cancer treated in the adjuvant lapatinib and/or trastuzumab Treatment Optimization Trial. British journal of cancer 2020;122:1453-1460.

36. İçli F, Altundağ K, Akbulut H, et al. Nine weeks versus 1 year adjuvant trastuzumab in patients with early breast cancer: an observational study by the Turkish Oncology Group (TOG). Breast Cancer 2015;22:480-485.

37. Narayan HK, French B, Khan AM, et al. Noninvasive Measures of Ventricular-Arterial Coupling and Circumferential Strain Predict Cancer Therapeutics–Related Cardiac Dysfunction. JACC: Cardiovascular Imaging 2016;9:1131-1141.

38. Grazziotin LR, Picon PD. Observational study of trastuzumab-related cardiotoxicity in early and metastatic breast cancer. Journal of Oncology Pharmacy Practice 2017;23:264-272.

39. Ulas A, Kos T, Avci N, et al. Patients with HER2-positive Early Breast Cancer Receiving Adjuvant Trastuzumab: Clinicopathological Features, Efficacy, and Factors Affecting Survival. Asian Pacific Journal of Cancer Prevention 2015;16:1643-1649.

40. Swain SM, Ewer MS, Viale G, et al. Pertuzumab, trastuzumab, and standard anthracycline- and taxane-based chemotherapy for the neoadjuvant treatment of patients with HER2-positive localized breast cancer (BERENICE): a phase II, open-label, multicenter, multinational cardiac safety study. Annals of Oncology 2018;29:646-653.

41. Choe JC, Choi JH, Choi JH, et al. Prolonged electromechanical delay as an early predictor of trastuzumab-induced cardiotoxicity in patients undergoing treatment for breast cancer. Clinical Cardiology 2018;41:1308-1314.

42. CHAN S, FRIEDRICHS K, YELLE L, et al. Prospective Randomized Trial of Docetaxel Versus Doxorubicin in Patients With Metastatic Breast Cancer. Journal of clinical oncology 1999;17:2341-2354.

43. Litvak A, Batukbhai B, Russell SD, et al. Racial disparities in the rate of cardiotoxicity of HER2-targeted therapies among women with early breast cancer. Cancer 2018;124:1904-1911.

44. Conduit C, de Boer RH, Lok S, et al. Real‐world impact of anti‐HER2 therapy‐related cardiotoxicity in patients with advanced HER2‐positive breast cancer. Asia-Pacific Journal of Clinical Oncology 2020;16:356-362.

45. Laufer-Perl M, Arnold JH, Mor L, et al. The association of reduced global longitudinal strain with cancer therapy-related cardiac dysfunction among patients receiving cancer therapy. Clinical Research in Cardiology 2020;109:255-262.

46. Pivot X, Pegram M, Cortes J, et al. Three-year follow-up from a phase 3 study of SB3 (a trastuzumab biosimilar) versus reference trastuzumab in the neoadjuvant setting for human epidermal growth factor receptor 2–positive breast cancer. European Journal of Cancer 2019;120:1-9.

47. van der Voort A, van Ramshorst MS, van Werkhoven ED, et al. Three-Year Follow-up of Neoadjuvant Chemotherapy With or Without Anthracyclines in the Presence of DualERBB2 Blockade in Patients WithERBB2 -Positive Breast Cancer. JAMA Oncology 2021;7:978.

48. Tarantini L, Cioffi G, Gori S, et al. Trastuzumab Adjuvant Chemotherapy and Cardiotoxicity in Real-World Women With Breast Cancer. Journal of Cardiac Failure 2012;18:113-119.

49. Piccart-Gebhart MJ, Procter M, Leyland-Jones B, et al. Trastuzumab after adjuvant chemotherapy in HER2-positive breast cancer. N Engl J Med 2005;353:1659-72.

50. Wadhwa D, Fallah-Rad N, Grenier D, et al. Trastuzumab mediated cardiotoxicity in the setting of adjuvant chemotherapy for breast cancer: a retrospective study. Breast Cancer Research and Treatment 2009;117:357-364.

51. Cardinale D, Colombo A, Torrisi R, et al. Trastuzumab-induced cardiotoxicity: clinical and prognostic implications of troponin I evaluation. J Clin Oncol 2010;28:3910-6.

52. Fried G, Regev T, Moskovitz M. Trastuzumab-related cardiac events in the treatment of early breast cancer. Breast Cancer Research and Treatment 2013;142:1-7.

53. Dodos F, Halbsguth T, Erdmann E, Hoppe UC. Usefulness of myocardial performance index and biochemical markers for early detection of anthracycline-induced cardiotoxicity in adults. Clinical Research in Cardiology 2008;97:318-326.
